# Excessive response to provocation rather than disinhibition mediates irritable behaviour in Huntington’s disease

**DOI:** 10.3389/fnins.2022.993357

**Published:** 2022-12-29

**Authors:** Duncan James McLauchlan, David E. J. Linden, Anne E. Rosser

**Affiliations:** ^1^Division of Psychological Medicine and Clinical Neurosciences, Cardiff University, Cardiff, United Kingdom; ^2^Department of Neurology, Morriston Hospital, Swansea Bay University Health Board, Swansea, United Kingdom; ^3^Cardiff University Brain Research Imaging Center, Cardiff University, Cardiff, United Kingdom; ^4^Faculty of Health, Medicine and Life Sciences, School for Mental Health and Neuroscience, Maastricht University, Maastricht, Netherlands; ^5^Brain Repair Group, School of Biosciences, Cardiff University, Cardiff, United Kingdom; ^6^Brain Repair and Intracranial Neurotherapeutics (B.R.A.I.N.) Biomedical Research Unit, College of Biomedical and Life Sciences, Cardiff University, Cardiff, United Kingdom

**Keywords:** Huntington’s disease, impulsivity, aggression, inhibition, neuropsychiatric disorders

## Abstract

**Background:**

Irritable and impulsive behaviour are common in Huntington’s disease (HD: an autosomal dominant disorder causing degeneration in cortico-striatal networks). However, the cognitive mechanisms underlying these symptoms remain unclear, and previous research has not determined if common mechanisms underpin both symptoms. Here we used established and novel tasks to probe different aspects of irritable and impulsive behaviour to determine the neural mechanisms involved.

**Methods:**

We recruited a cohort of 53 gene positive HD participants and 26 controls from non-affected family members and local volunteers. We used established questionnaire measures of irritability in HD (Snaith Irritability Scale, Problem Behaviours Assessment) and impulsivity [Urgency, Premeditation Perseverance, Sensation-seeking, Positive urgency scale (UPPSP), Barratt Impulsivity Scale], in addition to cognitive tasks of provocation, motor inhibition, delay discounting and decision making under uncertainty. We used generalised linear models to determine differences between cases and controls, and associations with irritability in the HD group.

**Results:**

We found differences between cases and controls on the negative urgency subscale of the UPPSP, which was associated with irritability in HD. The frustrative non-reward provocation task also showed differences between cases and controls, in addition to predicting irritability in HD. The stop signal reaction time task showed case-control differences but was not associated with irritability in HD. None of the other measures showed group differences or predicted irritability in HD after correcting for confounding variables.

**Discussion:**

Irritability in HD is mediated by excessive response to provocation, rather than a failure of motor inhibition.

## Background

Impulsivity [acting quickly without forethought or regard for potential consequences (Daruna et al., 1993; Evenden J., 1999; Dalley and Roiser, 2012)] and irritability [a psychological state characterised by impatience, intolerance and poorly controlled anger (Snaith et al., 1978; Snaith and Taylor, 1985)] are commonly found in a number of neurological conditions affecting the frontal lobes, basal ganglia and brainstem (Voon et al., 2010; Grassi et al., 2015; Lansdall et al., 2017; Perry et al., 2017). Impulsivity and irritability often co-occur, but each can also occur in isolation. Impulsive and irritable behaviour are both common in Huntington’s disease (HD: an autosomal dominant inherited neurodegenerative disease affecting cortico-striatal networks). Early clinical descriptions of HD emphasise aberrant behaviour, reporting frequent findings of alcohol dependency, impaired judgement, aggression and hypersexual behaviour (Spillane and Phillips, 1937; Dewhurst et al., 1969, 1970; Dewhurst, 1970; Huntington, 2003), whilst more recent work systematically captures increased levels of risk-taking in HD (McDonell et al., 2020). More systematic attempts to characterise irritability and aggression have found prevalence rates of between 38 and 73%, and an increase in these behaviours as the disease progresses through the pre-motor and early manifest stages (Berrios et al., 2001; Craufurd et al., 2001; van Duijn et al., 2007, 2014; Reedeker et al., 2012; Thompson et al., 2012). Furthermore, irritability and aggression form a common behavioural domain in HD (Craufurd et al., 2001; Paulsen et al., 2001; Rickards et al., 2011). Impulsivity has been less comprehensively studied, but impairments on both task and questionnaire measures of impulsive behaviour have been found in HD patients compared with healthy controls (Stout et al., 2001; Galvez et al., 2017; Johnson et al., 2017).

Impulsive and irritable behaviour also has significant consequences on function and quality of life for HD patients and carers (Ready et al., 2008; Carlozzi and Tulsky, 2013; Read et al., 2013), with irritable behaviour being found at higher rates in nursing home residents compared with those still managed at home (Shiwach, 1994; Wheelock et al., 2003). However, despite the prevalence of irritable and impulsive behaviour in HD, our knowledge of the contributory neuropsychological mechanisms remains limited, which in turn limits the development of targeted interventions.

Converging evidence in animals and humans has suggested that irritable and aggressive behaviour is mediated by two neural systems (Siegel et al., 1999; Gregg and Siegel, 2001; Nelson and Trainor, 2007; Lischinsky and Lin, 2020): (1) an ascending serotonergic network involving the periaqueductal grey and hypothalamus, that is modulated by thalamic, hippocampal, amygdala and anterior cingulate cortex connections governing response to omitted reward and threat; (2) an inhibitory network centred on the prefrontal cortex (in particular the inferior frontal gyrus: IFG) and the indirect pathway of the basal ganglia. There are several strands of evidence supporting a role of the serotonin system: work in healthy volunteers has shown increased aggressive behaviour on a cognitive task when tryptophan is acutely depleted (Moeller et al., 1996; Bjork et al., 1999); animal studies have demonstrated more aggressive behaviours with lesions of the periaqueductal grey, and hypothalamus (Gregg and Siegel, 2001); and studies of clinical populations prone to reactive aggression have most commonly reported lesions of the prefrontal cortex (Volkow et al., 1995; Best et al., 2002; Coccaro et al., 2007; Narayan et al., 2007), but have also shown altered activity or damage to the hypothalamus (Tonkonogy, 1991; George et al., 2004).

Impulsivity is also widely accepted to involve a number of contributory cognitive mechanisms: impaired motor inhibition (in common with irritability), valuing immediate over delayed reward, and an inability to learn or predict future rewards (Evenden, 1999; Aron et al., 2003b; Bari and Robbins, 2013; Robbins and Dalley, 2017). Imaging studies using cognitive tasks such as the stop signal reaction time task or GoNogo task have shown that the inferior frontal gyrus, and indirect pathway of the basal ganglia are central to motor inhibition (Aron et al., 2003a,^2007^). Learning and predicting future reward [measured using the Iowa gambling task (IGT)] is affected by damage to the amygdala and ventromedial prefrontal cortex (Bechara et al., 1997, 1999). A meta-analysis of functional imaging studies using delay-discounting tasks has shown increased activity in the striatum and medial prefrontal cortex (Wesley and Bickel, 2014) which is supported by lesion studies in animals that have shown increased valuation of immediate rewards over delayed rewards with lesions of the striatum, and also the basolateral amygdala (Cardinal et al., 2001; Winstanley et al., 2004).

Both the hypothalamus (Politis et al., 2008; Gabery et al., 2015), and corticostriatal networks (Vonsattel et al., 1985; Enzi et al., 2012) are well known to be affected by HD pathology. Furthermore, HD causes impairments in reinforcement learning and cognitive control (Lawrence et al., 1996, 1998). However, there are comparatively few previous studies of the neuro-cognitive basis of irritable and aggressive behaviour in HD: one study showed altered amygdala-prefrontal connectivity in a pre-manifest (before onset of motor symptoms) HD cohort, but no behavioural differences with controls on a provocation task (Klöppel et al., 2010). A further imaging study in pre-motor HD participants found an association between irritability scores and pulvinar perfusion during an anger induction task, but no group differences in experience of anger were noted (Van den Stock et al., 2015). A number of groups have compared HD patients to controls on some domains of impulsive behaviour, reporting differences on Iowa gambling task performance (Stout et al., 2001), motor inhibition (Stout et al., 2011) and questionnaire measures of impulsivity (Johnson et al., 2017). However, studies of delay discounting in HD have only been reported as conference abstracts (Doridam et al., 2014), or in animal models (Beste et al., 2008; Doridam et al., 2014; Rao et al., 2014; Tedford et al., 2015; Adjeroud et al., 2017; Galvez et al., 2017; Johnson et al., 2017). Previous work on impulsivity has also neglected to include salient confounding variables in the HD population such as cognitive impairment, premorbid IQ, and medication.

The present study uses cognitive tasks to probe the relative contributions of motor inhibition and response to provocation to irritable and aggressive behaviour in HD. Furthermore, this work adopts a comprehensive self-report and task-based approach to assess impulsivity in HD whilst accounting for relevant confounding factors such as cognitive impairment, medication, and premorbid IQ. Specifically, we used well-established instruments and tasks to measure known domains of impulsive behaviour (Evenden J., 1999): employing the UPPS-P (Whiteside and Lynam, 2001) and Barratt Impulsivity Scale (Patton et al., 1995) to measure self-report impulsivity. Motor inhibition was measured using the stop signal reaction time task (SSRT task (Verbruggen et al., 2008)). We used the Kirby instrument (Kirby and Maraković, 1996) to measure delay discounting and the Iowa Gambling Task [IGT (Bechara et al., 1997)] as a measure of cognitive impulsivity. The irritability tasks included tasks of provocation: the cognitive task previously described by Klöppel et al. (2010), and a variant of a well-established animal protocol (Gallup, 1965).

We hypothesised that as HD neuropathology involves frontal networks (Beste et al., 2008; Rao et al., 2014), and the hypothalamus (Petersén and Björkqvist, 2006; Politis et al., 2008) relatively early in the disease, but the ventral striatum is involved later (Vonsattel et al., 1985; Kassubek et al., 2004; Douaud et al., 2009) (1) both impaired inhibition and excessive response to provocation would contribute to irritability in HD and (2) HD patients would differ from control participants more markedly on motor inhibition and decision making under conditions of uncertainty, than on delay discounting.

## Materials and methods

### Ethical approval and data availability

All procedures in this study abided by the principles set out in the Declaration of Helsinki. Ethical approval was gained from the Research Ethics Committee for Wales (03/WA/0300). Suitably anonymised data is available from the authors on reasonable request.

### Participants and recruitment

All patient participants were recruited from the South Wales HD management clinic, which routinely has between 300 and 400 confirmed HD gene carriers under regular follow-up. 53 patient participants (disease stage–pre-motor onset to moderately affected) with a CAG repeat length >35 in the Huntingtin gene were recruited. Previous work (Thompson et al., 2012; McAllister et al., 2021) has indicated that irritability starts in the premanifest stage and peaks in the early manifest stage, hence the inclusion of HD participants without motor symptoms. Participants were aged 18 or over and free of any illness or injury potentially affecting brain function. Twenty-six control participants were recruited from HD family members not at risk of HD (either partners, carers, or spouses without a family history of HD, or confirmed gene negative status), and also through local advertising within Cardiff University. Participants were paid expenses (maximum €20), but were explicitly informed this was not dependent on task performance.

### Procedures

All participants completed a medical and medication history as well as a neurological examination using the total motor score (TMS) of the Unified Huntington’s Disease Rating Scale (UHDRS), if they had not already been examined using this tool in the preceding 3 months. Participants completed a phonemic verbal fluency task as a measure of cognitive impairment–a standard assessment of cognition in HD (Landwehrmeyer et al., 2016). This study was part of a wider research project into the cognitive basis of psychopathology in HD (McLauchlan, 2018), the total battery of 14 tasks also included tasks measuring motivation, learning, estimation and other cognitive processes hypothesised to contribute to apathy (McLauchlan et al., 2019) and depression (McLauchlan et al., 2022), although there is no overlap with the tasks and questionnaires presented in this work. The battery was administered in random order. Breaks between tasks were allowed *ad libitum* and the battery could be completed over 2 days for any participant who requested it. Participants had all task-specific features explained by the research team in person before starting each one, and practice levels were included for all tasks except the Frustrative non-reward task (owing to the nature of the process being tested).

### Questionnaires

Questionnaire assessments were completed prior to the cognitive tasks, to avoid bias on behalf of the rater.

### Irritability questionnaires

All participants completed the Snaith Irritability Score (SIS) and the short form of the Problem Behaviours Assessment (PBAs) which includes subscores for irritability and aggressive behaviour. The PBAs rates a number of domains of psychopathology in HD–these are scored for frequency and severity. The SIS and PBAs are well validated instruments, which are widely used for assessing irritability in HD (Snaith et al., 1978; Callaghan et al., 2015; Landwehrmeyer et al., 2016). The outcome measures were the total score of the SIS and the product of the severity and frequency scores in the irritability subdomain of the PBAs. Participants were classed as irritable if they scored >13 on the SIS or ≥4 on the PBAs irritability subscore.

### Impulsivity questionnaires

Participants completed two self-report questionnaire measures of impulsivity: the Urgency, Premeditation Perseverance, Sensation-seeking, Positive urgency scale (UPPSP) (Whiteside and Lynam, 2001) comprising subdomains of Negative Urgency (acting quickly under conditions of negative affect), lack of Premeditation (acting without forethought), lack of Perseverance (poor attention and/or persistence), Sensation-seeking (enjoying novel experiences or risk), and Positive Urgency (acting quickly under conditions of positive affect); and the Barrett Impulsiveness Scale (BIS) (Patton et al., 1995), which contains subdomains of motor (acting quickly), attention (focussing on current tasks) and non-planning (planning and thinking deliberately). The outcome measures were the total score of the BIS in addition to all subdomains, and the individual subdomains of the UPPSP.

### Impulsivity cognitive tasks

#### Kirby delay discounting instrument

This assessment asks participants to choose between two theoretical options–a smaller monetary amount available immediately, or a larger amount available after a time delay (Kirby and Maraković, 1996). Twenty-seven different options are presented. The outcome measure is the kD: the slope of the time-depreciation curve (Kaplan et al., 2016). Higher kD values indicate steeper discounting with time and hence higher impulsivity.

#### Stop signal reaction time task

This task is a well-established measure of inhibitory function (Aron et al., 2003a; Verbruggen et al., 2008). Participants were asked to respond to a visual stimulus by pressing a button on the keyboard as quickly as possible. On some trials, the visual stimulus was shortly followed by an auditory stimulus–on these trials participants had to withhold their response. Stop signal reaction time (the ability to withhold a prepotent response) was the outcome measure and was calculated automatically using standard methods (the average “go” reaction time minus the stop signal delay at which participants are successful on 50% of trials) (Verbruggen et al., 2008). Slower stop signal reaction time indicates poorer inhibitory function.

#### Iowa gambling task

This task is a measure of learning from outcome and decision making under uncertainty, that is associated with cognitive impulsivity (Bechara et al., 1997; Upton et al., 2011; Aram et al., 2019). Participants are asked to select from 4 packs of cards (A, B, C, and D) to maximise monetary reward and minimise loss. Every selection wins a monetary reward, but after some selections, losses also occur. Packs A and B have higher upfront gains, but over time they have larger losses outweighing these gains. The outcome measure was the number of selections from the disadvantageous packs (A and B) in the final 25 rounds of the game–this has been shown to be the most reliable outcome measure in a meta-analysis (Steingroever et al., 2013).

### Frustrative cognitive tasks

#### Kloppel task

This established task of frustration (Klöppel et al., 2010) asks participants to judge which is the larger of 2 squares, and gives inappropriately incorrect feedback on 14% of trials. There were 100 trials in total. Participants were asked to rate their feelings [from 0 (not experiencing the emotion at all) to 100 (highest intensity possible)] of anger, frustration and irritability before and after completing the task. These emotional ratings were interspersed with non-irritability related emotions to score in order to obscure the purpose of the task from participants. The outcome measure was the total score of anger, frustration and irritability after the task. Premature (responding before the screen terminated) and repeated responses (repetitive button pressing when asked for a response) were also included as outcome measures.

### Frustrative non-reward task

This novel task instructed participants to fill in a questionnaire of demographic details and set passwords. The participants would fill in demographic details (date of birth, address, and passwords) before an automated message would inform them that their data was not saved and would have to be re-entered. This automated message recurred twice before permitting the end of the data entry. Participants were informed of the need to enter demographic data and passwords before entering baseline emotion scores (of 0–100 for anger, frustration, and irritability) after the data had been entered had finished. The outcome measure was the total score of anger, frustration, and irritability after the task.

### Statistical analysis

All analyses were performed in R, a widely available open source software package (R Core Team, 2015). IQ scores were calculated using Crawford’s demographic method which has been shown to outperform alternative methods such as reading tests in HD populations (Crawford et al., 1988, 2001; Carlozzi et al., 2011). Neuroleptic and antidepressant medications were converted into olanzapine-equivalent and fluoxetine-equivalent doses using established methods (Leucht et al., 2014; Hayasaka et al., 2015). All variables were compared between groups using binomial models. Outcome measures were first compared between HD cases and controls, and subsequently outcome measures were compared between irritable and non-irritable HD cases. Medication, age, sex, cognitive impairment (verbal fluency score), full scale IQ, motor disability (TMS score), and reaction time (for the SSRT) were considered to be potential confounders. All binomial models were compared with and without potential confounders using likelihood ratio tests. Any confounders improving the original model were included in a final multi-variable model.

## Results

### Demographics

Huntington’s disease participants did not differ from the control group on age, or gender balance (Table 1). HD participants had significantly lower IQ score, lower verbal fluency score, had more motor impairment and higher doses of anti-psychotic and antidepressant medications. 20 of the 53 HD participants were irritable (>13 on the SIS or ≥4 on the PBAs irritability subscore), whilst 0 of the 26 control participants were irritable.

**TABLE 1:** Demographics.

	HD	Controls
Age	52 (33–82)	52.5 (20–75)
Gender (Male)	27/53	9/26
Premanifest status	12/53	–
FSIQ	103.55 (88.75–125.27)	109.73 (89.79–128.51)
Olanzapine equivalent dose (mg)	1.92 (0–41.25)	0
Fluoxetine equivalent dose (mg)	22.27 (0–146.5)	2.4 (0–22.2)
CAG repeat length	42.5 (38–50)	–
UHDRS total motor score	35.49 (0–89)	1.48 (0–6)
Verbal fluency score	29.29 (5–52)	45.46 (16–75)
Irritable	20/53	0/26
Aggressive	14/53	0/26

Differences on demographic variables between Huntington’s disease and control participants, showing mean and range. UHDRS, Unified Huntington’s Disease Rating Scale; FSIQ, full scale intelligence quotient. Olanzapine and fluoxetine equivalent doses calculated from meta-analyses 92, 93. Participants were defined as Irritable if they scored >4 on the Problem Behaviours Assessment (PBAs) irritability subscore and aggressive if they scored >4 on the PBAs aggression subscore.

### Measures of impulsivity

#### Urgency, premeditation perseverance, sensation-seeking, positive urgency scale

**FIGURE 1:**
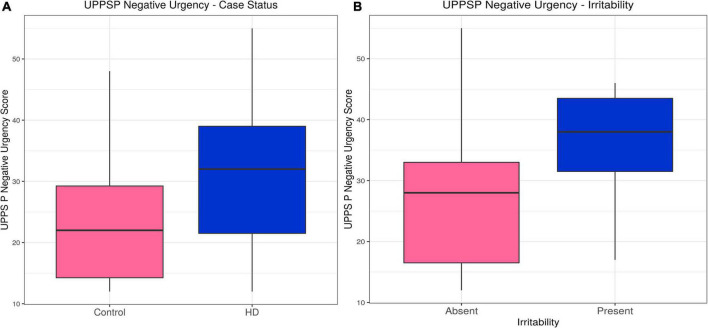
UPPS P Negative Urgency–Effects of case status and irritability status. Box and whisker plots (median, interquartile range, and outliers as individual points). **(A)** Group differences between Huntington’s (HD) cases and control participants. **(B)** Group differences between irritable and non-irritable HD cases. UPPS P, Urgency (Negative), (lack) Premeditation, (lack) Perseverance, Sensation Seeking, Positive Urgency.

Huntington’s disease participants had higher scores on the Negative Urgency (acting impulsively under conditions of negative affect) subscale (estimate = 0.23, *p* = 1.50 × 10^–6^). Likelihood ratio tests for potential confounding variables suggested gender, IQ and medication improved the model, but their inclusion did not alter the association between case status and Negative Urgency score (estimate = 0.17, *p* = 0.0016). Within the HD group, irritability status (SIS > 13 or ≥4 PBAs irritability) was associated with higher Negative Urgency score (estimate = 0.092, *p* = 0.0071), an association that did not change with inclusion of the only significant confounding variable, the verbal fluency score (estimate = 0.024, *p* = 0.0077). The subscales for Lack of Perseverance and Lack of Premeditation did not show any significant differences between HD patients and controls, nor was there any association with irritability in the HD group once statistically significant confounding variables were included in the models. HD participants had lower scores on the Sensation Seeking subscale when significant confounders (age, gender, IQ, anti-psychotic dose and verbal fluency score) were included in the model (estimate = −0.15, *p* = 0.029), and there was no association with irritability in the HD group. No associations were found between Positive Urgency score and HD status, or Positive Urgency score and irritability in the HD group when significant confounding variables were included in the models. In sum, Negative Urgency was the only subscale on which we found both a group difference and association with irritability in HD participants (see Table 2, Supplementary Tables 1A–J, Figure 1, and Supplementary Figure 1).

**TABLE 2:** Questionnaire and task scores.

	HD	Controls
UPPS P negative urgency	30.37 (12–55)	24.19 (12–48)
UPPS P lack of premeditiation	21.25 (0–42)	19.46 (11–44)
UPPS P lack of perseverance	21.55 (10–37)	17.85 (10–37)
UPPS P sensation seeking	27.75 (12–60)	28.5 (12–48)
UPPS P positive urgency	38.88 (0–56)	49.08 (14–56)
BIS total score	66.42 (0–108)	59.27 (32–110)
BIS attention sub-score	17.71 (8–29)	14.69 (8–32)
BIS motor sub-score	24.96 (12–40)	22.42 (11–37)
BIS non-planning sub-score	26.35 (11–44)	22.15 (11–44)
Delay discounting (kD)	0.08 (0–0.25)	0.06 (0–0.25)
SSRT (ms)	496.25 (113.9–1901.7)	304.42 (225.5–392.7)
IGT (maximum 25)	5.78 (0–15)	3.73 (0–12)
Kloppel post-task irritability score (maximum 300)	77.49 (0–300)	55.27 (0–180)
Kloppel premature response	20.73 (0–83)	9.91 (0–75)
Kloppel total responses	100.54 (11–457)	109.27 (99–181)
FNR post-task irritability score (maximum 300)	79.48 (0–300)	29.17 (0–110)

Differences on questionnaire and task scores between Huntington’s disease and control participants, showing mean and range. UPPS P, Urgency (Negative), (lack) Premeditation, (lack) Perseverance, Sensation Seeking, Positive Urgency; BIS, Barratt Impulsiveness Scale; SSRT, stop signal reaction time; IGT, Iowa gambling task; FNR, frustrative non-reward.

#### Barratt impulsivity scale

Huntington’s disease participants had higher total Barratt Impulsivity Scale (BIS) scores than controls, and their BIS scores showed an association with irritability. However, neither of these associations were significant with inclusion of relevant confounders in the models (estimate = 0.049, *p* = 0.16) and (estimate = 0.057, *p* = 0.15), respectively. Similar to the results seen in the BIS Total score, BIS Attention scores were higher in the HD group (consistent with worse attention in the HD cohort) and showed an association with irritability status in the HD group before inclusion of relevant confounding variables. However, neither of these associations was maintained when relevant confounders were included in the models (estimate = 0.099, *p* = 0.17) and (estimate = 0.24, *p* = 0.13). The BIS Non-Planning and Motor subscales showed significantly higher scores in HD participants compared with controls that did not survive inclusion of relevant confounders in the models. Neither subscale showed an association with irritability in the HD group. In sum, there was no group difference and no association with irritability for any BIS component when confounders were included in the model (see Table 2, Supplementary Tables 2A–H, and Supplementary Figure 2).

### Impulsivity tasks

#### Kirby delay discounting instrument

No differences were seen in kD (slope of the discounting curve) between HD cases and controls, and no association was seen with irritability in the HD group (see Table 2, Supplementary Tables 3A,B, and Supplementary Figures 3A,B).

#### Stop signal reaction time task

**FIGURE 2:**
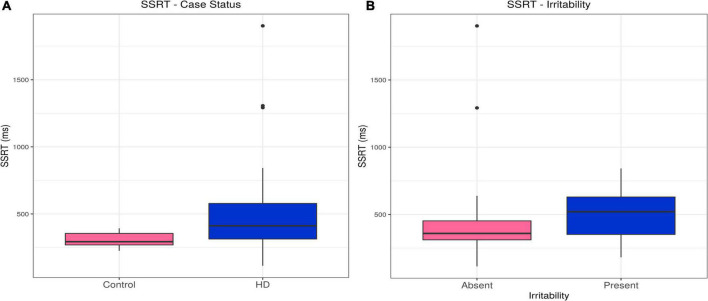
SSRT–Effects of case status and irritability status. Box and whisker plots (median, interquartile range, and outliers as individual points). **(A)** Group differences between Huntington’s (HD) cases and control participants. **(B)** Group differences between irritable and non-irritable HD cases. SSRT, stop signal reaction time.

Huntington’s disease cases had slower stop signal reaction times (SSRTs) than controls (estimate = 191.84, *p* = 0.00054). This difference persisted, even with inclusion of relevant confounders in the model (olanzapine equivalent dose, reaction time; estimate = 82.64, *p* = 0.025). There was no association between SSRT and irritability in the HD group (see Table 2, Supplementary Tables 4A,B, and Figures 2A,B).

#### Iowa gambling task

Huntington’s disease participants made more frequent selections from disadvantageous decks than control participants (estimate = 0.44, *p* = 0.00024). However, this relationship did not survive inclusion of confounding variables in the model. No association between task performance and irritability was found in the HD group (see Table 2, Supplementary Tables 5A,B, and Supplementary Figures 3C,D).

In sum, only the SSRT showed group differences between HD and control participants, and none of the impulsivity tasks showed an association with irritability in HD after inclusion of confounders.

### Frustrative tasks

#### Kloppel task

Huntington’s disease participants had higher post task irritability scores than controls following the task (estimate = 0.34, *p* = < 2 × 10^–16^). This difference was maintained, even when relevant confounding variables were included in the model (estimate = 0.22, *p* = 0.00048). *However, no association was seen with irritability status in the HD group*. HD participants made premature responses more frequently than controls, but this association was reversed with inclusion of confounding variables in the model (estimate = −0.46, *p* = 0.0075). No association between premature responses and irritability was found in the HD group. No difference was found between HD cases and controls in total number of responses when the models included relevant confounding variables. Furthermore, no association was found between total responses and irritability in the HD group (see Table 2, Supplementary Tables 6A–F, and Supplementary Figures 4A–F).

#### Frustrative non-reward task

**FIGURE 3:**
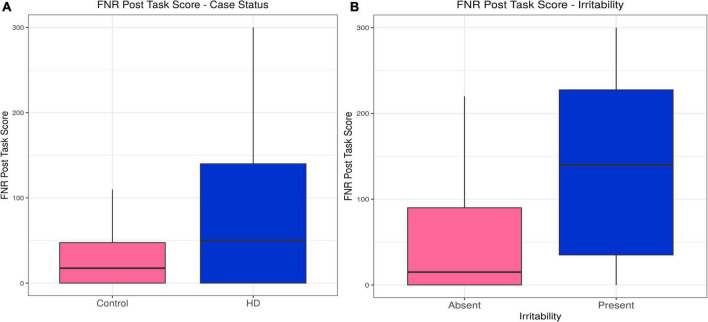
Frustrative non-reward (FNR)–Effects of case status and irritability status. Box and whisker plots (median, interquartile range, and outliers as individual points). **(A)** Group differences between Huntington’s (HD) cases and control participants. **(B)** Group differences between irritable and non-irritable HD cases.

Huntington’s disease participants reported higher levels of irritability following the frustrative non-reward (FNR) task than controls, a relationship that was unaffected by inclusion of confounding variables in the model (estimate = 0.54, *p* = 3.81 × 10^–9^). Irritability scores following the FNR task correlated with irritability status in the HD group (estimate = 0.011, *p* = 0.031), and the model was not improved by any confounding variable (see Table 2, Supplementary Tables 7A,B, and Figures 3A,B).

In sum, HD participants showed higher post-task irritability on both tasks, but only the irritability evoked by the FNR task was associated with irritability status in binomial models.

## Discussion

This comprehensive investigation of cognitive and motivational mechanisms of irritability has brought several novel insights into the mechanisms underlying altered behaviour and psychological reactions of participants with HD. HD participants were more sensitive to provocation than controls, as seen on the frustrative non-reward task. In keeping with previous findings, HD participants also demonstrated deficits in inhibition on the SSRT compared with controls, even after correction for potentially confounding variables. However, only the excessive response to provocation, and not the inhibitory deficit, was associated with irritability (measured on the SIS and PBAs) in HD. In line with previous findings on impulsive behaviour in HD, we also found deficits on tasks of decision making under ambiguity (IGT), and the Barrett Impulsivity Scale compared to controls, but these differences were not maintained after correction for confounding variables. Finally, in contrast to studies in animal models, we did not find deficits in delay discounting in HD.

Although irritability in HD is a complex construct (Simpson et al., 2019; Dale et al., 2022), our findings suggest a major cognitive mechanism underlying irritable and aggressive behaviour in HD is represented by excessive response to provocation. This mechanism is mediated by the core aggression circuit which includes the periaqueductal grey (PAG), thalamus, hypothalamus and amygdala; and is regulated by frontal structures which exert top-down inhibition (anterior cingulate cortex, orbitofrontal cortex and medial prefrontal cortex) and guided by learned aggression networks which incentivise aggressive behaviour under favourable environmental conditions (striatum, ventral tegmental area) (reviewed in Lischinsky et al. (Lischinsky and Lin, 2020)). HD neuropathology affects both the frontal inhibitory regions (Hobbs et al., 2010; Thu et al., 2010; Rao et al., 2014) and core aggression circuit (Kassubek et al., 2005; Gabery et al., 2015; Ahveninen et al., 2018), whilst impairments of the core cognitive functions of these regions; les (emotion recognition and moderation, inhibition) are central clinical features of HD (Stout et al., 2011; Tabrizi et al., 2013). Previous imaging and pathology studies on irritable and aggressive behaviour in HD patients, have shown associations between irritability and reduced grey matter volume in the pulvinar, the striatum (Martinez-Horta et al., 2021), and the globus pallidus (Singh-Bains et al., 2016); and pulvinar activity during anger induction. In a group of premanifest HD gene carriers (but not healthy control participants) self-reported irritability during a provocation task showed positive correlation with BOLD signal in the amygdala and negative correlation with BOLD signal in the orbito-frontal cortex (Klöppel et al., 2010). Collectively the neurobiological evidence in HD suggests alterations in a network mediating response to threat rather than brain regions involved in inhibition such as the inferior frontal gyrus or subthalamic nucleus. This supports our findings of irritability in HD being mediated by excess response to provocation rather than impaired inhibition.

However, we also found some evidence for impaired inhibition in HD. Whilst previous groups have found differences between HD participants and healthy control participants on a number of dimensions of impulsivity (Stout et al., 2001; Rao et al., 2014; Galvez et al., 2017; Johnson et al., 2017), the only consistent group differences in our study were deficits of inhibition on the SSRT and acting impulsively under conditions of negative affect (Negative Urgency from the UPPSP). The differences in inhibition persisted even with the inclusion of reaction time and anti-psychotic dose in the model. Impairment in inhibitory function in HD has been a consistent finding in both motor manifest and premanifest HD participants (Beste et al., 2008; Rao et al., 2014). The indirect pathway of the basal ganglia is known to be selectively damaged early in HD (Albin et al., 1992; Schroll et al., 2015), this pathway contributes to inhibitory function (Schroll et al., 2015), and hence its degeneration may contribute to failure of inhibition seen in HD. Although previous studies found deficits in decision making under conditions of ambiguity in HD (Stout et al., 2001; Adjeroud et al., 2017), we were not able to replicate these after accounting for relevant confounders. Previous studies had smaller numbers of HD participants, and did not include medication and other potential confounders in their analyses. Although studies in animal models of HD (Tedford et al., 2015) have found deficits in delay discounting tasks, we did not replicate this in human subjects. Delay discounting is mediated by the ventral striatum and medial prefrontal cortex (McClure et al., 2004; Frost and McNaughton, 2017). As neurodegeneration in HD is known to progress in a dorsal to ventral direction through the striatum (Vonsattel et al., 1985), one explanation for our failure to replicate the animal data may relate to disease stage; specifically that the animal models may have modelled a later disease stage than that present in the participants of our study. Whilst previous work has reported evidence of increased self-reported impulsivity on the BIS (Johnson et al., 2017) in HD participants, we did not replicate this finding when confounders were included in the model. This may reflect the lack of correction for confounders in previous work or may relate to the difficulties with the use of self-report scales in a disease that causes anosognosia (Ho et al., 2006). Impulsive behaviour has been associated with altered dopaminergic inputs to the striatum. In particular, dopamine antagonists have been shown to improve motor inhibition on the SSRT in animals, whilst 18F Fallypride PET studies of dopaminergic binding have shown reductions in D2 receptor binding in the dorsal striatum is associated with reduced inhibition on the SSRT (Dalley and Robbins, 2017). In keeping with this, anti-psychotic treatments have level IV evidence for managing some aspects of disinhibited behaviour in HD patients (Tibrewal et al., 2017; Karagas et al., 2020; Rossi and Oh, 2020).

This work has some limitations. First, although the verbal fluency task that we used as a measure of cognitive decline does have extensive evidence of dysfunction in HD (Ho et al., 2002; Stout et al., 2011; Landwehrmeyer et al., 2016), showing differences from controls and longitudinal progression in premanifest and motor manifest participants, the symbol digit modality task and Stroop task show impairments earlier in the disease course and may be more sensitive to change in HD (Stout et al., 2011, 2014). Secondly, this study is part of a wider research project into the cognitive basis of neuropsychiatric dysfunction in HD (McLauchlan, 2018; McLauchlan et al., 2019) and a total of 14 tasks were completed. Although we randomised task order for each participant, completing these tasks as part of the entire battery may have artificially accentuated irritability. One aspect of impulsivity not tested in our battery was decision-making under risk, however, previous studies in HD have not found deficits in this process (Adjeroud et al., 2017; Galvez et al., 2017). In keeping with established practice in HD cognitive and behavioural research (Tabrizi et al., 2013; Landwehrmeyer et al., 2016), we attempted to account for the altered neuropsychiatric milieu experienced by HD families and therefore more accurately assess the effect of HD neuropathology on cognitive processes, by preferentially recruiting controls from gene negative family members. However, the control sample did include some participants not from a HD family potentially reducing this efficacy of this approach. We included participants both with and without motor onset and controlled for this by including UHDRS TMS in models of outcome. However, it is well-established that HD has a prodromal phase with behavioural changes [including irritability (Epping et al., 2016)] and cognitive deficits occurring before motor onset (Tabrizi et al., 2022). Despite this, we cannot entirely exclude the possibility that the processes underlying irritability change with disease stage. Finally we did consider the possibility that performing any cognitive task may be somewhat frustrating for participants (and hence act as a provocation task); thus self-report irritability perhaps should have been measured after all of the tasks. However, the post-task self-report irritability ratings only correlated with formal irritability assessment (on the SIS and PBAs) on the FNR task.

In summary, this work has demonstrated that whilst HD participants do have both inhibitory deficits, and excessive response to provocation compared to controls, irritability in HD is only associated with provocation. As the provocation response is mediated by reduced tone in a predominantly serotonergic network [the core aggression circuit (Moeller et al., 1996; Bjork et al., 1999)] this may explain why serotonergic drug treatments can be effective in treating irritability in HD (van Duijn, 2010). As a consequence, carers and family members may find it helpful to avoid provocation and adopt a ‘pick your battles’ approach when managing irritable behaviour in patients with HD. Furthermore, although previous studies have reported that HD neuropathology causes impairments in a range of cognitive processes associated with impulsive behaviour, the only consistent behavioural deficit in relation to impulsivity was seen in a slowed inhibitory response.

## Data availability statement

Information on the data underpinning the results presented here, including how to access them, can be found in the Cardiff University data catalogue at: http://research.cardiff.ac.uk.

## Ethics statement

The studies involving human participants were reviewed and approved by Research Ethics Committee for Wales (03/WA/0300). The patients/participants provided their written informed consent to participate in this study.

## Author contributions

DM: research project—conception, organization, execution; statistical analysis—design, execution; manuscript preparation—writing of the first draft. DL and AER: research project—conception, organization; statistical analysis—review and critique; manuscript preparation—review and critique. All authors contributed to the article and approved the submitted version.

## References

[B1] Adjeroud N. Besnard J. Verny C. Prundean A. Scherer C. Gohier B. (2017). Dissociation between decision-making under risk and decision-making under ambiguity in premanifest and manifest Huntington’s disease. *Neuropsychologia* 103 87–95. 10.1016/j.neuropsychologia.2017.07.011 28712946

[B2] Ahveninen L. M. Stout J. C. Georgiou-Karistianis N. Lorenzetti V. Glikmann-Johnston Y. (2018). Reduced amygdala volumes are related to motor and cognitive signs in Huntington’s disease: The IMAGE-HD study. *Neuroimage Clin.* 18 881–887. 10.1016/j.nicl.2018.03.027 29876272 PMC5988225

[B3] Albin R. L. Reiner A. Anderson K. D. Dure L. S. IV Handelin B. Balfour R. (1992). Preferential loss of striato-external pallidal projection neurons in presymptomatic Huntington’s disease. *Ann. Neurol.* 31 425–430. 10.1002/ana.410310412 1375014

[B4] Aram S. Levy L. Patel J. B. Anderson A. A. Zaragoza R. Dashtestani H. (2019). The iowa gambling task: A review of the historical evolution, scientific basis, and use in functional neuroimaging. *Sage Open* 9:21582440198 56911.

[B5] Aron A. R. Durston S. Eagle D. M. Logan G. D. Stinear C. M. Stuphorn V. (2007). Converging evidence for a fronto-basal-ganglia network for inhibitory control of action and cognition. *J. Neurosci.* 27 11860–11864. 10.1523/JNEUROSCI.3644-07.2007 17978025 PMC6673355

[B6] Aron A. R. Watkins L. Sahakian B. J. Monsell S. Barker R. A. Robbins T. W. (2003b). Task-set switching deficits in early-stage Huntington’s disease: Implications for basal ganglia function. *J. Cogn. Neurosci.* 15 629–642. 10.1162/089892903322307357 12965037

[B7] Aron A. R. Fletcher P. C. Bullmore E. T. Sahakian B. J. Robbins T. W. (2003a). Stop-signal inhibition disrupted by damage to right inferior frontal gyrus in humans. *Nat. Neurosci.* 6 115–116. 10.1038/nn1003 12536210

[B8] Bari A. Robbins T. W. (2013). Inhibition and impulsivity: Behavioral and neural basis of response control. *Prog. Neurobiol.* 108 44–79. 10.1016/j.pneurobio.2013.06.005 23856628

[B9] Bechara A. Damasio H. Damasio A. R. Lee G. P. (1999). Different contributions of the human amygdala and ventromedial prefrontal cortex to decision-making. *J. Neurosci. Off. J. Soc. Neurosci.* 19 5473–5481. 10.1523/JNEUROSCI.19-13-05473.1999 10377356 PMC6782338

[B10] Bechara A. Damasio H. Tranel D. Damasio A. R. (1997). Deciding advantageously before knowing the advantageous strategy. *Science* 275 1293–1295. 10.1126/science.275.5304.1293 9036851

[B11] Berrios G. E. Wagle A. C. Marková I. S. Wagle S. A. Ho L. W. Rubinsztein D. C. (2001). Psychiatric symptoms and CAG repeats in neurologically asymptomatic Huntington’s disease gene carriers. *Psychiatry Res.* 102 217–225. 10.1016/S0165-1781(01)00257-8 11440772

[B12] Best M. Williams J. M. Coccaro E. F. (2002). Evidence for a dysfunctional prefrontal circuit in patients with an impulsive aggressive disorder. *Proc. Natl. Acad. Sci. U.S.A.* 99 8448–8453. 10.1073/pnas.112604099 12034876 PMC123087

[B13] Beste C. Saft C. Andrich J. Gold R. Falkenstein M. (2008). Response inhibition in Huntington’s disease-a study using ERPs and sLORETA. *Neuropsychologia* 46 1290–1297. 10.1016/j.neuropsychologia.2007.12.008 18241897

[B14] Bjork J. M. Dougherty D. M. Moeller F. G. Cherek D. R. Swann A. C. (1999). The effects of tryptophan depletion and loading on laboratory aggression in men: Time course and a food-restricted control. *Psychopharmacology (Berl.)* 142 24–30. 10.1007/s002130050858 10102779

[B15] Burdick J. D. Roy A. L. Raver C. C. (2013). Evaluating the Iowa gambling task as a direct assessment of impulsivity with low-income children. *Personal. Individ. Differ.* 55 771–776. 10.1016/j.paid.2013.06.009 24072950 PMC3780341

[B16] Callaghan J. Stopford C. Arran N. Boisse M. F. Coleman A. Santos R. D. (2015). Reliability and factor structure of the short problem behaviors assessment for Huntington’s disease (PBA-s) in the TRACK-HD and REGISTRY studies. *J. Neuropsychiatry Clin. Neurosci.* 27 59–64. 10.1176/appi.neuropsych.13070169 25716488

[B17] Cardinal R. N. Pennicott D. R. Sugathapala C. L. Robbins T. W. Everitt B. J. (2001). Impulsive choice induced in rats by lesions of the nucleus accumbens core. *Science* 292 2499–2501. 10.1126/science.1060818 11375482

[B18] Carlozzi N. E. Stout J. C. Mills J. A. Duff K. Beglinger L. J. Aylward E. H. (2011). Estimating premorbid IQ in the prodromal phase of a neurodegenerative disease. *Clin. Neuropsychol.* 25 757–777. 10.1080/13854046.2011.577811 21660882 PMC3159182

[B19] Carlozzi N. E. Tulsky D. S. (2013). Identification of health-related quality of life (HRQOL) Issues Relevant to Individuals with HD. *J. Health Psychol.* 18 212–225. 10.1177/1359105312438109 22427174 PMC3643297

[B20] Coccaro E. F. McCloskey M. S. Fitzgerald D. A. Phan K. L. (2007). Amygdala and orbitofrontal reactivity to social threat in individuals with impulsive aggression. *Biol. Psychiatry* 62 168–178. 10.1016/j.biopsych.2006.08.024 17210136

[B21] Craufurd D. Thompson J. C. Snowden J. S. (2001). Behavioral changes in Huntington Disease. *Neuropsychiatry Neuropsychol. Behav. Neurol.* 14 219–226. 11725215

[B22] Crawford J. R. Millar J. Milne A. B. (2001). Estimating premorbid IQ from demographic variables: A comparison of a regression equation vs. clinical judgement. *Br. J. Clin. Psychol.* 40(Pt 1) 97–105. 10.1348/014466501163517 11317952

[B23] Crawford J. R. Parker D. M. Besson J. A. (1988). Estimation of premorbid intelligence in organic conditions. *Br. J. Psychiatry J. Ment. Sci.* 153 178–181. 10.1192/bjp.153.2.178 2978378

[B24] Dale M. Wood A. Zarotti N. Eccles F. Gunn S. Kiani R. (2022). Using a clinical formulation to understand psychological distress in people affected by Huntington’s disease: A descriptive, evidence-based model. *J. Pers. Med.* 12:1222. 10.3390/jpm12081222 35893316 PMC9332789

[B25] Dalley J. W. Robbins T. W. (2017). Fractionating impulsivity: Neuropsychiatric implications. *Nat. Rev. Neurosci.* 18 158–171. 10.1038/nrn.2017.8 28209979

[B26] Dalley J. W. Roiser J. P. (2012). Dopamine, serotonin and impulsivity. *Neuroscience* 215 42–58. 10.1016/j.neuroscience.2012.03.065 22542672

[B27] Daruna J. H. Barnes P. A. Daruna J. H. Barnes P. A. (1993). *A neurodevelopmental view of impulsivity.* Available online at: https://www.scienceopen.com/document?vid=65ded95b-cb83-4e0d-807c-e4ad100bb23e (accessed March 15, 2018).

[B28] Dewhurst K. (1970). Personality disorder in Huntington’s disease. *Psychiatr. Clin. (Basel)* 3 221–229. 10.1159/000278607 4248176

[B29] Dewhurst K. Oliver J. Trick K. L. McKnight A. L. (1969). Neuro-psychiatric aspects of Huntington’s disease. *Confin. Neurol.* 31 258–268. 10.1159/000103486 4243863

[B30] Dewhurst K. Oliver J. E. McKnight A. L. (1970). Socio-psychiatric consequences of Huntington’s disease. *Br. J. Psychiatry J. Ment. Sci.* 116 255–258. 10.1192/bjp.116.532.255 4244788

[B31] Doridam J. Roussel M. Simonin C. Benoist A. Tir M. Godefroy O. (2014). H14 evaluation of impulsivity in Huntington’s disease with a delay discounting task. *J. Neurol. Neurosurg. Psychiatry* 85 (Suppl. 1) A56–A56. 10.1136/jnnp-2014-309032.159

[B32] Douaud G. Behrens T. E. Poupon C. Cointepas Y. Jbabdi S. Gaura V. (2009). In vivo evidence for the selective subcortical degeneration in Huntington’s disease. *Neuroimage* 46 958–966. 10.1016/j.neuroimage.2009.03.044 19332141

[B33] Enzi B. Edel M.-A. Lissek S. Peters S. Hoffmann R. Nicolas V. (2012). Altered ventral striatal activation during reward and punishment processing in premanifest Huntington’s disease: A functional magnetic resonance study. *Exp. Neurol.* 235 256–264. 10.1016/j.expneurol.2012.02.003 22366326

[B34] Epping E. A. Kim J.-I. Craufurd D. Brashers-Krug T. M. Anderson K. E. McCusker E. (2016). Longitudinal psychiatric symptoms in prodromal Huntington’s disease: A decade of data. *Am. J. Psychiatry* 173 184–192. 10.1176/appi.ajp.2015.14121551 26472629 PMC5465431

[B35] Evenden J. (1999). Impulsivity: A discussion of clinical and experimental findings. *J. Psychopharmacol. Oxf. Engl.* 13 180–192. 10.1177/026988119901300211 10475725

[B36] Evenden J. L. (1999). Varieties of impulsivity. *Psychopharmacology (Berl.)* 146 348–361. 10.1007/PL00005481 10550486

[B37] Frost R. McNaughton N. (2017). The neural basis of delay discounting: A review and preliminary model. *Neurosci. Biobehav. Rev.* 79 48–65. 10.1016/j.neubiorev.2017.04.022 28465167

[B38] Gabery S. Georgiou-Karistianis N. Lundh S. H. Cheong R. Y. Churchyard A. Chua P. (2015). Volumetric analysis of the hypothalamus in Huntington disease using 3T MRI: The IMAGE-HD Study. *PLoS One* 10:e0117593. 10.1371/journal.pone.0117593 25659157 PMC4319930

[B39] Gallup G. G. (1965). Aggression in rats as a function of frustrative nonreward in a straight alley. *Psychon. Sci.* 3 99–100. 10.3758/BF03343040

[B40] Galvez V. Fernandez-Ruiz J. Bayliss L. Ochoa-Morales A. Hernandez-Castillo C. R. Díaz R. (2017). Early Huntington’s disease: Impulse control deficits but correct judgment regarding risky situations. *J. Huntingtons Dis.* 6 73–78. 10.3233/JHD-160223 28339399

[B41] George D. T. Rawlings R. R. Williams W. A. Phillips M. J. Fong G. Kerich M. (2004). A select group of perpetrators of domestic violence: Evidence of decreased metabolism in the right hypothalamus and reduced relationships between cortical/subcortical brain structures in position emission tomography. *Psychiatry Res.* 130 11–25. 10.1016/S0925-4927(03)00105-7 14972365

[B42] Grassi G. Pallanti S. Righi L. Figee M. Mantione M. Denys D. (2015). Think twice: Impulsivity and decision making in obsessive–compulsive disorder. *J. Behav. Addict.* 4 263–272. 10.1556/2006.4.2015.039 26690621 PMC4712760

[B43] Gregg T. R. Siegel A. (2001). Brain structures and neurotransmitters regulating aggression in cats: Implications for human aggression. *Prog. Neuropsychopharmacol. Biol. Psychiatry* 25 91–140. 10.1016/s0278-5846(00)00150-0 11263761

[B44] Hayasaka Y. Purgato M. Magni L. R. Ogawa Y. Takeshima N. Cipriani A. (2015). Dose equivalents of antidepressants: Evidence-based recommendations from randomized controlled trials. *J. Affect. Disord.* 180 179–184. 10.1016/j.jad.2015.03.021 25911132

[B45] Ho A. K. Robbins A. O. G. Barker R. A. (2006). Huntington’s disease patients have selective problems with insight. *Mov. Disord. Off. J. Mov. Disord. Soc.* 21 385–389. 10.1002/mds.20739 16211608

[B46] Ho A. K. Sahakian B. J. Robbins T. W. Barker R. A. Rosser A. E. Hodges J. R. (2002). Verbal fluency in Huntington’s disease: A longitudinal analysis of phonemic and semantic clustering and switching. *Neuropsychologia* 40 1277–1284. 10.1016/s0028-3932(01)00217-2 11931930

[B47] Hobbs N. Z. Henley S. M. D. Ridgway G. R. Wild E. J. Barker R. A. Scahill R. I. (2010). The progression of regional atrophy in premanifest and early Huntington’s disease: A longitudinal voxel-based morphometry study. *J. Neurol. Neurosurg. Psychiatry* 81 756–763. 10.1136/jnnp.2009.190702 19955112

[B48] Huntington G. (2003). On chorea. George Huntington, M.D. *J. Neuropsychiatry Clin. Neurosci.* 15 109–112. 10.1176/jnp.15.1.109 12556582

[B49] Johnson P. L. Potts G. F. Sanchez-Ramos J. Cimino C. R. (2017). Self-reported impulsivity in Huntington’s disease patients and relationship to executive dysfunction and reward responsiveness. *J. Clin. Exp. Neuropsychol.* 39 694–706. 10.1080/13803395.2016.1257702 27892808

[B50] Kaplan B. Amlung M. Reed D. Jarmolowicz D. P. McKerchar T. L. Lemley S. M. (2016). Automating scoring of delay discounting for the 21- and 27-item monetary choice questionnaires. *Behav. Anal.* 39 293–304. 10.1007/s40614-016-0070-9 31976983 PMC6701266

[B51] Karagas N. E. Rocha N. P. Stimming E. F. (2020). Irritability in Huntington’s disease. *J. Huntingt. Dis.* 9 107–113. 10.3233/JHD-200397 32417789 PMC7369067

[B52] Kassubek J. Juengling F. D. Ecker D. Landwehrmeyer G. B. (2005). Thalamic atrophy in Huntington’s disease co-varies with cognitive performance: A morphometric MRI analysis. *Cereb. Cortex* 15 846–853. 10.1093/cercor/bhh185 15459079

[B53] Kassubek J. Juengling F. D. Kioschies T. Henkel K. Karitzky J. Kramer B. (2004). Topography of cerebral atrophy in early Huntington’s disease: A voxel based morphometric MRI study. *J. Neurol. Neurosurg. Psychiatry* 75 213–220. 14742591 PMC1738932

[B54] Kirby K. N. Maraković N. N. (1996). Delay-discounting probabilistic rewards: Rates decrease as amounts increase. *Psychon. Bull. Rev.* 3 100–104. 10.3758/BF03210748 24214810

[B55] Klöppel S. Stonnington C. M. Petrovic P. Mobbs D. Tüscher O. Craufurd D. (2010). Irritability in pre-clinical Huntington’s disease. *Neuropsychologia* 48 549–557. 19878688 10.1016/j.neuropsychologia.2009.10.016 PMC2809920

[B56] Landwehrmeyer G. B. Fitzer-Attas C. J. Giuliano J. D. Gonçalves N. Anderson K. E. Cardoso F. (2016). Data analytics from enroll-HD, a global clinical research platform for Huntington’s disease. *Mov. Disord. Clin. Pract.* 4 212–224. 10.1002/mdc3.12388 30363395 PMC6174428

[B57] Lansdall C. J. Coyle-Gilchrist I. T. S. Jones P. S. Vázquez Rodríguez P. Wilcox A. Wehmann E. (2017). Apathy and impulsivity in frontotemporal lobar degeneration syndromes. *Brain J. Neurol.* 140 1792–1807. 10.1093/brain/awx101 PMC5868210 28486594

[B58] Lawrence A. D. Hodges J. R. Rosser A. E. Kershaw A. ffrench-Constant C. Rubinsztein D. C. (1998). Evidence for specific cognitive deficits in preclinical Huntington’s disease. *Brain J. Neurol.* 121(Pt 7) 1329–1341. 10.1093/brain/121.7.1329 9679784

[B59] Lawrence A. D. Sahakian B. J. Hodges J. R. Rosser A. E. Lange K. W. Robbins T. W. (1996). Executive and mnemonic functions in early Huntington’s disease. *Brain J. Neurol.* 119(Pt 5) 1633–1645. 10.1093/brain/119.5.1633 8931586

[B60] Leucht S. Samara M. Heres S. Patel M. X. Woods S. W. Davis J. M. (2014). Dose equivalents for second-generation antipsychotics: The minimum effective dose method. *Schizophr. Bull.* 40 314–326. 10.1093/schbul/sbu001 24493852 PMC3932104

[B61] Lischinsky J. E. Lin D. (2020). Neural mechanisms of aggression across species. *Nat. Neurosci.* 23 1317–1328. 10.1038/s41593-020-00715-2 33046890 PMC13152643

[B62] Martinez-Horta S. Sampedro F. Horta-Barba A. Perez-Perez J. Pagonabarraga J. Gomez-Anson B. (2021). Structural brain correlates of irritability and aggression in early manifest Huntington’s disease. *Brain Imaging Behav.* 15 107–113. 10.1007/s11682-019-00237-x 31898092

[B63] McAllister B. Gusella J. F. Landwehrmeyer G. B. Lee J. M. MacDonald M. E. Orth M. (2021). Timing and impact of psychiatric, cognitive, and motor abnormalities in huntington disease. *Neurology* 96 e2395–e2406. 10.1212/WNL.0000000000011893 33766994 PMC8166441

[B64] McClure S. M. Laibson D. I. Loewenstein G. Cohen J. D. (2004). Separate neural systems value immediate and delayed monetary rewards. *Science* 306 503–507. 10.1126/science.1100907 15486304

[B65] McDonell K. E. Ciriegio A. E. Pfalzer A. C. Hale L. Shiino S. Riordan H. (2020). Risk-taking behaviors in Huntington’s disease. *J. Huntingtons Dis.* 9 359–369. 10.3233/JHD-200431 33164940

[B66] McLauchlan D. (2018). *Objective assessment of the neuropsychiatric symptoms in Huntington’s Disease*. Ph.D. thesis. Cardiff: Cardiff University.

[B67] McLauchlan D. Lancaster T. Craufurd D. Linden D. E. J. Rosser A. E. (2022). Different depression: Motivational anhedonia governs antidepressant efficacy in Huntington’s disease. *Brain Commun.* 4:fcac278. 10.1093/braincomms/fcac278 36440100 PMC9683390

[B68] McLauchlan D. J. Lancaster T. Craufurd D. Linden D. E. J. Rosser A. E. (2019). Insensitivity to loss predicts apathy in Huntington’s disease. *Mov. Disord. Off. J. Mov. Disord. Soc.* 34 1381–1391. 10.1002/mds.27787 31361357

[B69] Moeller F. G. Dougherty D. M. Swann A. C. Collins D. Davis C. M. Cherek D. R. (1996). Tryptophan depletion and aggressive responding in healthy males. *Psychopharmacology (Berl.)* 126 97–103. 10.1007/BF02246343 8856827

[B70] Narayan V. M. Narr K. L. Kumari V. Woods R. P. Thompson P. M. Toga A. W. (2007). Regional cortical thinning in subjects with violent antisocial personality disorder or schizophrenia. *Am. J. Psychiatry* 164 1418–1427. 17728428 10.1176/appi.ajp.2007.06101631 PMC3197838

[B71] Nelson R. J. Trainor B. C. (2007). Neural mechanisms of aggression. *Nat. Rev. Neurosci.* 8 536–546. 10.1038/nrn2174 17585306

[B72] Patton J. H. Stanford M. S. Barratt E. S. (1995). Factor structure of the Barratt Impulsiveness Scale. *J. Clin. Psychol.* 51 768–774. 10.1002/1097-4679(199511)51:6<768::AID-JCLP2270510607>3.0.CO;2-1 8778124

[B73] Paulsen J. S. Ready R. E. Hamilton J. M. Mega M. S. Cummings J. L. (2001). Neuropsychiatric aspects of Huntington’s disease. *J. Neurol. Neurosurg. Psychiatry* 71 310–314. 10.1136/jnnp.71.3.310 11511702 PMC1737562

[B74] Perry D. C. Datta S. Sturm V. E. Wood K. A. Zakrzewski J. Seeley W. W. (2017). Reward deficits in behavioural variant frontotemporal dementia include insensitivity to negative stimuli. *Brain J. Neurol.* 140 3346–3356. 10.1093/brain/awx259 29053832 PMC5841034

[B75] Petersén A. Björkqvist M. (2006). Hypothalamic-endocrine aspects in Huntington’s disease. *Eur. J. Neurosci.* 24 961–967. 10.1111/j.1460-9568.2006.04985.x 16925587

[B76] Politis M. Pavese N. Tai Y. F. Tabrizi S. J. Barker R. A. Piccini P. (2008). Hypothalamic involvement in Huntington’s disease: An in vivo PET study. *Brain J. Neurol.* 131(Pt 11) 2860–2869. 10.1093/brain/awn244 18829696

[B77] R Core Team (2015). *R: A language and environment for statistical computing.* Vienna: R Foundation for Statistical Computing.

[B78] Rao J. A. Harrington D. L. Durgerian S. Reece C. Mourany L. Koenig K. (2014). Disruption of response inhibition circuits in prodromal Huntington disease. *Cortex J. Devoted Study Nerv. Syst. Behav.* 58 72–85. 10.1016/j.cortex.2014.04.018 24959703 PMC4227536

[B79] Read J. Jones R. Owen G. Leavitt B. R. Coleman A. Roos R. A. (2013). Quality of life in Huntington’s disease: A comparative study investigating the impact for those with pre-manifest and early manifest disease, and their partners. *J. Huntingtons Dis.* 2 159–175. 10.3233/JHD-130051 25063513

[B80] Ready R. E. Mathews M. Leserman A. Paulsen J. S. (2008). Patient and caregiver quality of life in Huntington’s disease. *Mov. Disord. Off. J. Mov. Disord. Soc.* 23 721–726. 10.1002/mds.21920 18175350 PMC3789516

[B81] Reedeker N. Bouwens J. A. Giltay E. J. Le Mair S. E. Roos R. A. van der Mast R. C. (2012). Irritability in Huntington’s disease. *Psychiatry Res.* 200 813–818. 10.1016/j.psychres.2012.03.041 22537721

[B82] Rickards H. De Souza J. van Walsem M. van Duijn E. Simpson S. A. Squitieri F. (2011). Factor analysis of behavioural symptoms in Huntington’s disease. *J. Neurol. Neurosurg. Psychiatry* 82 411–412. 10.1136/jnnp.2009.181149 20392980

[B83] Robbins T. W. Dalley J. W. (2017). “Chapter 7–Impulsivity, risky choice, and impulse control disorders: Animal models,” in *Decision Neuroscience*, eds Dreher J.-C. Tremblay L. (San Diego, CA: Academic Press), 81–93. 10.1016/B978-0-12-805308-9.00007-5

[B84] Rossi G. Oh J. C. (2020). Management of Agitation in Huntington’s disease: A review of the literature. *Cureus* 12:e9748. 10.7759/cureus.9748 PMC7489774 32944463

[B85] Schroll H. Beste C. Hamker F. H. (2015). Combined lesions of direct and indirect basal ganglia pathways but not changes in dopamine levels explain learning deficits in patients with Huntington’s disease. *Eur. J. Neurosci.* 41 1227–1244. 10.1111/ejn.12868 25778633

[B86] Shiwach R. (1994). Psychopathology in Huntington’s disease patients. *Acta Psychiatr. Scand.* 90 241–246. 10.1111/j.1600-0447.1994.tb01587.x 7831992

[B87] Siegel A. Roeling T. A. Gregg T. R. Kruk M. R. (1999). Neuropharmacology of brain-stimulation-evoked aggression. *Neurosci. Biobehav. Rev.* 23 359–389. 10.1016/s0149-7634(98)00040-2 9989425

[B88] Simpson J. Dale M. Theed R. Gunn S. Zarotti N. Eccles F. J. R. (2019). Validity of irritability in Huntington’s disease: A scoping review. *Cortex J. Devoted Study Nerv. Syst. Behav.* 120 353–374. 10.1016/j.cortex.2019.06.012 31401402

[B89] Singh-Bains M. K. Tippett L. J. Hogg V. M. Synek B. J. Roxburgh R. H. Waldvogel H. J. (2016). Globus pallidus degeneration and clinicopathological features of Huntington disease. *Ann. Neurol.* 80 185–201. 10.1002/ana.24694 27255697

[B90] Snaith R. P. Constantopoulos A. A. Jardine M. Y. McGuffin P. (1978). A clinical scale for the self-assessment of irritability. *Br. J. Psychiatry J. Ment. Sci.* 132 164–171. 10.1192/bjp.132.2.164 623950

[B91] Snaith R. P. Taylor C. M. (1985). Irritability: Definition, assessment and associated factors. *Br. J. Psychiatry J. Ment. Sci.* 147 127–136. 10.1192/bjp.147.2.127 3840045

[B92] Spillane J. Phillips R. (1937). Huntington’s chorea in South Wales. *QJM Int. J. Med.* 6 403–423.

[B93] Steingroever H. Wetzels R. Horstmann A. Neumann J. Wagenmakers E. J. (2013). Performance of healthy participants on the Iowa gambling task. *Psychol. Assess.* 25 180–193. 22984804 10.1037/a0029929

[B94] Stout J. C. Paulsen J. S. Queller S. Solomon A. C. Whitlock K. B. Campbell J. C. (2011). Neurocognitive signs in prodromal Huntington disease. *Neuropsychology* 25 1–14. 10.1037/a0020937 20919768 PMC3017660

[B95] Stout J. C. Queller S. Baker K. N. Cowlishaw S. Sampaio C. Fitzer-Attas C. (2014). HD-CAB investigators. HD-CAB: A cognitive assessment battery for clinical trials in Huntington’s disease 1,2,3. *Mov. Disord.* 29 1281–1288. 10.1002/mds.25964 25209258

[B96] Stout J. C. Rodawalt W. C. Siemers E. R. (2001). Risky decision making in Huntington’s disease. *J. Int. Neuropsychol. Soc.* 7 92–101. 11253845 10.1017/s1355617701711095

[B97] Tabrizi S. J. Scahill R. I. Owen G. Durr A. Leavitt B. R. Roos R. A. (2013). Predictors of phenotypic progression and disease onset in premanifest and early-stage Huntington’s disease in the TRACK-HD study: Analysis of 36-month observational data. *Lancet Neurol.* 12 637–649. 10.1016/S1474-4422(13)70088-7 23664844

[B98] Tabrizi S. J. Schobel S. Gantman E. C. Mansbach A. Borowsky B. Konstantinova P. (2022). A biological classification of Huntington’s disease: The integrated staging system. *Lancet Neurol.* 21 632–644. 10.1016/S1474-4422(22)00120-X 35716693

[B99] Tedford S. E. Persons A. L. Napier T. C. (2015). Dopaminergic lesions of the dorsolateral striatum in rats increase delay discounting in an impulsive choice task. *PLoS One* 10:e0122063. 10.1371/journal.pone.0122063 25927685 PMC4415807

[B100] Thompson J. C. Harris J. Sollom A. C. Stopford C. L. Howard E. Snowden J. S. (2012). Longitudinal evaluation of neuropsychiatric symptoms in Huntington’s disease. *J. Neuropsychiatry Clin. Neurosci.* 24 53–60. 10.1176/appi.neuropsych.11030057 22450614

[B101] Thu D. C. V. Oorschot D. E. Tippett L. J. Nana A. L. Hogg V. M. Synek B. J. (2010). Cell loss in the motor and cingulate cortex correlates with symptomatology in Huntington’s disease. *Brain J. Neurol.* 133(Pt 4) 1094–1110. 10.1093/brain/awq047 20375136

[B102] Tibrewal P. Bastiampillai T. Dhillon R. Cheng R. Fonseka H. T. (2017). Use of zuclopenthixol in the treatment of aggression in Huntington’s disease. *Asian J. Psychiatry* 26 152–153. 10.1016/j.ajp.2017.01.011 28483083

[B103] Tonkonogy J. M. (1991). Violence and temporal lobe lesion: Head CT and MRI data. *J. Neuropsychiatry Clin. Neurosci.* 3 189–196. 10.1176/jnp.3.2.189 1821235

[B104] Upton D. J. Bishara A. J. Ahn W.-Y. Stout J. C. (2011). Propensity for risk taking and trait impulsivity in the Iowa gambling task. *Personal. Individ. Differ.* 50 492–495. 10.1016/j.paid.2010.11.013 PMC3042697 21359157

[B105] Van den Stock J. De Winter F.-L. Ahmad R. Sunaert S. Van Laere K. Vandenberghe W. (2015). Functional brain changes underlying irritability in premanifest Huntington’s disease. *Hum. Brain Mapp.* 36 2681–2690. 10.1002/hbm.22799 25858294 PMC6869704

[B106] van Duijn E. (2010). Treatment of Irritability in Huntington’s Disease. *Curr. Treat. Options Neurol.* 12 424–433. 10.1007/s11940-010-0088-3 20730109 PMC2918785

[B107] van Duijn E. Craufurd D. Hubers A. A. M. Giltay E. J. Bonelli R. Rickards H. (2014). Neuropsychiatric symptoms in a European Huntington’s disease cohort (REGISTRY). *J. Neurol. Neurosurg. Psychiatry* 85 1411–1418. 10.1136/jnnp-2013-307343 24828898

[B108] van Duijn E. Kingma E. M. van der Mast R. C. (2007). Psychopathology in verified Huntington’s disease gene carriers. *J. Neuropsychiatry Clin. Neurosci.* 19 441–448. 10.1176/jnp.2007.19.4.441 18070848

[B109] Verbruggen F. Logan G. D. Stevens M. A. (2008). STOP-IT: Windows executable software for the stop-signal paradigm. *Behav. Res. Methods* 40 479–483. 10.3758/brm.40.2.479 18522058

[B110] Volkow N. D. Tancredi L. R. Grant C. Gillespie H. Valentine A. Mullani N. (1995). Brain glucose metabolism in violent psychiatric patients: A preliminary study. *Psychiatry Res.* 61 243–253. 10.1016/0925-4927(95)02671-j 8748468

[B111] Vonsattel J. P. Myers R. H. Stevens T. J. Ferrante R. J. Bird E. D. Richardson E. P. Jr. (1985). Neuropathological classification of Huntington’s disease. *J. Neuropathol. Exp. Neurol.* 44 559–577. 10.1097/00005072-198511000-00003 2932539

[B112] Voon V. Reynolds B. Brezing C. Gallea C. Skaljic M. Ekanayake V. (2010). Impulsive choice and response in dopamine agonist-related impulse control behaviors. *Psychopharmacology (Berl.)* 207 645–659. 10.1007/s00213-009-1697-y 19838863 PMC3676926

[B113] Wesley M. J. Bickel W. K. (2014). Remember the Future II: Meta-analyses and functional overlap of working memory and delay discounting. *Biol. Psychiatry* 75 435–448. 10.1016/j.biopsych.2013.08.008 24041504 PMC3943930

[B114] Wheelock V. L. Tempkin T. Marder K. Nance M. Myers R. H. Zhao H. (2003). Predictors of nursing home placement in Huntington disease. *Neurology* 60 998–1001. 10.1212/01.WNL.0000052992.58107.67 12654967

[B115] Whiteside S. P. Lynam D. R. (2001). The Five Factor Model and impulsivity: Using a structural model of personality to understand impulsivity. *Personal. Individ. Differ.* 30 669–689. 10.1016/S0191-8869(00)00064-7

[B116] Winstanley C. A. Theobald D. E. H. Cardinal R. N. Robbins T. W. (2004). Contrasting roles of basolateral amygdala and orbitofrontal cortex in impulsive choice. *J. Neurosci. Off. J. Soc. Neurosci.* 24 4718–4722. 10.1523/JNEUROSCI.5606-03.2004 15152031 PMC6729470

