# Supplementary material for: Excessive response to provocation rather than disinhibition mediates irritable behaviour in Huntington’s disease
Source: Front Neurosci. 2022 Dec 29;16:993357. doi: 10.3389/fnins.2022.993357 (PMC9836783; doi:10.3389/fnins.2022.993357)
Supplement: Supplementary file 1 [file Data_Sheet_1.pdf]

Supplementary Table 1a: UPPS P Negative Urgency - Case Status

	Estimate	P Value
(Intercept)	3.19	$<2\text{x}10^{-16}$
Case Status	0.23	1.50×10^{-6}
(Intercept)	4.28	$<2\text{x}10^{-16}$
Case Status	0.17	0.0016
Gender (Male)	-0.23	6.58×10^{-7}
FSIQ	-0.0094	1.32×10^{-5}
Olanzapine Equivalent Dose (mg)	0.0046	0.33
Fluoxetine Equivalent Dose (mg)	0.00048	0.66

Influence of case status (HD vs control) on UPPS P Negative Urgency score. Pseudo $R^2 = 0.55$, standard error 2.059. UPPS P - Urgency(Negative), (lack) Premeditation, (lack) Perseverance, Sensation Seeking, Positive Urgency. FSIQ - full scale intelligence quotient.

Supplementary Table 1b: Irritability - UPPS P Negative Urgency

	Estimate	P Value
(Intercept)	-3.22	0.0049
UPPS P Negative Urgency Score	0.092	0.0071
(Intercept)	0.34	0.34
UPPS P Negative Urgency Score	0.024	0.0077
Verbal Fluency	-0.021	0.0052

Influence of UPPS P Negative Urgency score on Irritability status in Huntington's disease cases. Pseudo $R^2 = 0.76$, standard error 2.059. UPPS P - Urgency(Negative), (lack) Premeditation, (lack) Perseverance, Sensation Seeking, Positive Urgency. FSIQ - full scale intelligence quotient.

Supplementary Table 1c: UPPS P Lack of Premeditation - Case Status

	Estimate	P Value
(Intercept)	2.97	$<2\text{x}10^{-16}$
Case Status	0.088	0.10
(Intercept)	4.13	$<2\text{x}10^{-16}$
Case Status	0.010	0.87
Age	-0.00051	0.78
Gender (Male)	-0.058	0.28
FSIQ	-0.010	6.49×10^{-5}
Olanzapine Equivalent Dose (mg)	0.019	0.00036
Fluoxetine Equivalent Dose (mg)	-0.0015	0.26

Influence of case status (HD vs control) on UPPS P lack of premeditation score. Pseudo $R^2 = 0.38$, standard error 1.073. UPPS P - Urgency(Negative), (lack) Premeditation, (lack) Perseverance, Sensation Seeking, Positive Urgency. FSIQ - full scale intelligence quotient.

Supplementary Table 1d: Irritability - UPPS P Lack of Premeditation

	Estimate	P Value
(Intercept)	-2.025	0.043
UPPS P Lack of Premeditation	0.078	0.075
(Intercept)	0.38	0.87
UPPS P Lack of Premeditation	0.19	0.12
Verbal Fluency	-0.17	0.017

Influence of UPPS P lack of premeditation score on Irritability status in Huntington's disease cases. Pseudo $R^2 = 0.62$, standard error 0.96. UPPS P - Urgency(Negative), (lack) Premeditation, (lack) Perseverance, Sensation Seeking, Positive Urgency.

Supplementary Table 1e: UPPS P Lack of Perseverance - Case Status

	Estimate	P Value
(Intercept)	2.88	$<2x10^{-16}$
Case Status	0.19	0.00066
(Intercept)	3.87	$<2x10^{-16}$
Case Status	0.10	0.090
FSIQ	-0.0088	0.00045

Influence of case status (HD vs control) on UPPS P lack of perseverance score. Pseudo $R^2 = 0.39$, standard error 1.73. UPPS P - Urgency(Negative), (lack) Premeditation, (lack) Perseverance, Sensation Seeking, Positive Urgency. FSIQ - full scale intelligence quotient.

Supplementary Table 1f: Irritability - UPPS P Lack of Perseverance

	Estimate	P Value
(Intercept)	-1.97	0.035
UPPS P Lack of Perseverance	0.075	0.063
(Intercept)	1.63	0.47
UPPS P Lack of Perseverance	0.089	0.29
Verbal Fluency	-0.14	0.018

Influence of UPPS P lack of perseverance score on Irritability status in Huntington's disease cases. Pseudo $R^2 = 0.54$, standard error 1.022. UPPS P - Urgency(Negative), (lack) Premeditation, (lack) Perseverance, Sensation Seeking, Positive Urgency.

Supplementary Table 1g: UPPS P Sensation Seeking - Case Status

	Estimate	P Value
(Intercept)	3.35	$<2x10^{-16}$
Case Status	-0.027	0.55
(Intercept)	3.20	$<2x10^{-16}$
Case Status	-0.15	0.029
Age	-0.0076	3.64×10^{-5}
Gender (Male)	0.26	1.65×10^{-5}
FSIQ	0.0072	0.014
Olanzapine Equivalent Dose (mg)	0.015	0.45
Verbal Fluency	-0.0091	0.00030

Influence of case status (HD vs control) on UPPS P sensation seeking score. Pseudo $R^2 = 0.66$, standard error 1.89. UPPS P - Urgency(Negative), (lack) Premeditation, (lack) Perseverance, Sensation Seeking, Positive Urgency. FSIQ - full scale intelligence quotient.

Supplementary Table 1h: Irritability - UPPS P Sensation Seeking

	Estimate	P Value
(Intercept)	-0.48	0.54
UPPS P Sensation Seeking	0.0052	0.84
(Intercept)	2.012	0.28
UPPS P Sensation Seeking	0.079	0.20
Verbal Fluency	-0.16	0.023

Influence of UPPS P sensation seeking score on Irritability status in Huntington's disease cases. Pseudo $R^2 = 0.57$, standard error 1.00. UPPS P - Urgency(Negative), (lack) Premeditation, (lack) Perseverance, Sensation Seeking, Positive Urgency.

Supplementary Table 1i: UPPS P Positive Urgency - Case Status

	Estimate	P Value
(Intercept)	3.89	$<2x10^-16$
Case Status	-0.23	8.69×10^{-11}
(Intercept)	3.44	$<2x10^-16$
Case Status	-0.039	0.49
Age	-0.0036	0.011
FSIQ	0.0037	0.098
Olanzapine Equivalent Dose (mg)	-0.018	0.32
Fluoxetine Equivalent Dose (mg)	-0.00037	0.77
Verbal Fluency	0.0050	0.0068

Influence of case status (HD vs control) on UPPS P positive urgency score. Pseudo $R^2 = 0.49$, standard error 1.91. UPPS P - Urgency(Negative), (lack) Premeditation, (lack) Perseverance, Sensation Seeking, Positive Urgency. FSIQ - full scale intelligence quotient.

Supplementary Table 1j: Irritability - UPPS P Positive Urgency

	Estimate	P Value
(Intercept)	0.90	0.31
UPPS P Positive Urgency	-0.032	0.14
(Intercept)	5.89	0.033
UPPS P Positive Urgency	-0.057	0.20
Verbal Fluency	-0.14	0.025

Influence of UPPS P sensation seeking score on Irritability status in Huntington's disease cases. Pseudo R^2 = 0.56, standard error 1.01. UPPS P - Urgency(Negative), (lack) Premeditation, (lack) Perseverance, Sensation Seeking, Positive Urgency.

Supplementary Table 2a: BIS Total Score - Case Status

	Estimate	P Value
(Intercept)	4.082	$<2x10^{-16}$
Case Status	0.11	0.00019
(Intercept)	5.68	$<2x10^{-16}$
Case Status	0.049	0.16
Age	-0.0051	$5.82x10^{-7}$
FSIQ	-0.012	$<2x10^{-16}$
Olanzapine Equivalent Dose (mg)	0.0098	0.0013
Fluoxetine Equivalent Dose (mg)	-0.000059	0.93

Influence of case status (HD vs control) on Barratt Impulsiveness Scale total score. Pseudo R^2 = 0.72, standard error 2.45. FSIQ - full scale intelligence quotient.

Supplementary Table 2b: Irritability - BIS Total Score

	Estimate	P Value
(Intercept)	-3.18	0.017
BIS Total Score	0.041	0.027
(Intercept)	-0.32	0.91
BIS Total Score	0.057	0.15
Verbal Fluency	-0.15	0.028

Influence of Barratt Impulsiveness Scale total score on Irritability status in Huntington's disease cases. Pseudo R^2 = 0.59, standard error 0.98.

Supplementary Table 2c: BIS Attention Sub-score - Case Status

	Estimate	P Value
(Intercept)	2.69	$<2\text{x}10^{-16}$
Case Status	0.19	0.0022
(Intercept)	4.24	$<2\text{x}10^{-16}$
Case Status	0.099	0.17
Age	-0.0052	0.013
Gender (Male)	0.00087	0.99
FSIQ	-0.012	$1.83\text{x}10^{-5}$
Olanzapine Equivalent Dose (mg)	0.010	0.086
Fluoxetine Equivalent Dose (mg)	0.00058	0.69

Influence of case status (HD vs control) on Barratt Impulsiveness Scale attention sub-score. Pseudo $R^2 = 0.44$, standard error 1.35. FSIQ - full scale intelligence quotient.

Supplementary Table 2d: Irritability - BIS Attention Sub-score

	Estimate	P Value
(Intercept)	-2.94	0.0098
BIS Attention Sub-score	0.15	0.017
(Intercept)	2.19	0.53
BIS Attention Sub-score	0.24	0.13
Gender (Male)	-3.59	0.066
Verbal Fluency	-0.17	0.053

Influence of Barratt Impulsiveness Scale attention sub-score on Irritability status in Huntington's disease cases. Pseudo $R^2 = 0.70$, standard error 0.91.

Supplementary Table 2e: BIS Motor Sub-score - Case Status

	Estimate	P Value
(Intercept)	3.11	$<2\text{x}10^{-16}$
Case Status	0.11	0.032
(Intercept)	3.67	$<2\text{x}10^{-16}$
Case Status	-0.0052	0.94
FSIQ	-0.0037	0.24
Olanzapine Equivalent Dose (mg)	0.012	0.57
Verbal Fluency	-0.0032	0.20

Influence of case status (HD vs control) on Barratt Impulsiveness Scale motor sub-score. Pseudo $R^2 = 0.15$, standard error 1.58. FSIQ - full scale intelligence quotient.

Supplementary Table 2f: Irritability BIS Motor Sub-score

	Estimate	P Value
(Intercept)	-1.98	0.080
BIS Motor Sub-score	0.065	0.13
(Intercept)	0.423	0.85
BIS Motor Sub-score	0.15	0.12
Verbal Fluency	-0.16	0.029

Influence of Barratt Impulsiveness Scale motor sub-score on Irritability status in Huntington's disease cases. Pseudo $R^2 = 0.62$, standard error 0.96.

Supplementary Table 2g: BIS Non-Planning Sub-score - Case Status

	Estimate	P Value
(Intercept)	3.098	$<2x10^{-16}$
Case Status	0.17	0.00049
(Intercept)	4.92	$<2x10^{-16}$
Case Status	0.12	0.10
FSIQ	-0.016	8.69×10^{-8}
Olanzapine Equivalent Dose (mg)	0.0055	0.78
Verbal Fluency	-0.00041	0.87

Influence of case status (HD vs control) on Barratt Impulsiveness Scale non-planning sub-score. Pseudo $R^2 = 0.58$, standard error 1.60. FSIQ - full scale intelligence quotient.

Supplementary Table 2h: Irritability BIS Non-Planning Sub-score

	Estimate	P Value
(Intercept)	-2.0022	0.043
BIS Non-Planning Sub-score	0.063	0.073
(Intercept)	1.19	0.63
BIS Non-Planning Sub-score	0.080	0.24
Verbal Fluency	-0.14	0.024

Influence of Barratt Impulsiveness Scale non-planning sub-score on Irritability status in Huntington's disease cases. Pseudo $R^2 = 0.55$, standard error 1.01.

Supplementary Table 3a: Delay Discounting - Case Status

	Estimate	P Value
(Intercept)	17.88	8.94×10^{-5}
Case Status	-5.40	0.27
(Intercept)	-7.92	0.74
Case Status	-1.81	0.64
Age	-0.29	0.047
Gender (Male)	-7.15	0.099
FSIQ	0.43	0.051

Influence of case status (HD vs control) on Delay discounting. Pseudo $R^2 = 0.11$, standard error 1.50. FSIQ - full scale intelligence quotient.

Supplementary Table 3b: Irritability - Delay Discounting

	Estimate	P Value
(Intercept)	-0.45	0.27
kD	-0.11	0.97
(Intercept)	3.51	0.049
kD	-3.11	0.64
Verbal Fluency	-0.13	0.023

Influence of Delay discounting on Irritability status in Huntington's disease cases. Pseudo $R^2 = 0.55$, standard error 1.07.

Supplementary Table 4a: SSRT - Case Status

	Estimate	P Value
(Intercept)	304.42	9.03×10^{-14}
Case Status	191.84	0.00054
(Intercept)	57.99	0.33
Case Status	82.64	0.025
Olanzapine Equivalent Dose (mg)	14.32	0.10
Reaction Time (ms)	0.40	$2.83 x 10^{-5}$

Influence of case status (HD vs control) on stop signal reaction time (SSRT). Pseudo $R^2 = 0.42$, standard error 0.39.

Supplementary Table 4b: Irritability - SSRT

	Estimate	P Value
(Intercept)	-0.74	0.21
SSRT	0.00048	0.64
(Intercept)	4.91	0.17
SSRT	-0.00010	0.98
Reaction Time (ms)	-0.0033	0.39
Verbal Fluency	-0.10	0.17

Influence of stop signal reaction time (SSRT) on Irritability status in Huntington's disease cases. Pseudo R^2 = 0.45, standard error 1.09.

Supplementary Table 5a: IGT Score - Case Status

	Estimate	P Value
(Intercept)	1.32	$<2x10^{-16}$
Case Status	0.44	0.00024
(Intercept)	2.46	0.0030
Case Status	-0.12	0.49
Age	0.014	0.0054
FSIQ	-0.013	0.099
Verbal Fluency	-0.0096	0.13

Influence of case status (HD vs control) on stop signal reaction time (SSRT). Pseudo R^2 = 0.31, standard error 1.62.

Supplementary Table 5b: Irritability IGT Score

	Estimate	P Value
(Intercept)	-0.59	0.31
IGT Score	0.034	0.67
(Intercept)	6.89	0.069
IGT Score	-0.24	0.44
Verbal Fluency	-0.24	0.030

Influence of Iowa gambling task (IGT) score on Irritability status in Huntington's disease cases. Pseudo R^2 = 0.65, standard error 0.93.

Supplementary Table 6a: Kloppel Post Task Score - Case Status

	Estimate	P Value
(Intercept)	4.01228	$<2x10^{-16}$
Case Status	0.33783	$<2x10^{-16}$
(Intercept)	2.41	$<2x10^{-16}$
Case Status	0.22	0.00048
Gender (Male)	-0.48	$<2x10^{-16}$
FSIQ	0.017	$4.42x10^{-11}$
Olanzapine Equivalent Dose (mg)	-0.29	$5.76x10^{-10}$
Fluoxetine Equivalent Dose (mg)	-0.015	$2.21x10^{-15}$
Verbal Fluency	-0.0010	0.61

Influence of case status (HD vs control) on Kloppel post task score. Pseudo R^2 = 0.87, standard error 9.90. FSIQ - full scale intelligence quotient

Supplementary Table 6b: Irritability - Kloppel Post Task Score

	Estimate	P Value
(Intercept)	-0.99	0.040
Kloppel Post-Task Irritability Score	0.0059	0.13
(Intercept)	1.58	0.93
Kloppel Post-Task Irritability Score	0.0029	0.87
FSIQ	0.068	0.75
Verbal Fluency	-0.35	0.12

Influence of Kloppel post task score on Irritability status in Huntington's disease cases. Pseudo R^2 = 0.82, standard error 0.74. FSIQ - full scale intelligence quotient

Supplementary Table 6c: Kloppel Premature Response - Case Status

	Estimate	P Value
(Intercept)	2.29345	$<2x10^{-16}$
Case Status	0.73812	$<2x10^{-16}$
(Intercept)	3.63	$3.31x10^{-10}$
Case Status	-0.46	0.00013
Age	0.0062	0.062
FSIQ	0.0071	0.19
Fluoxetine Equivalent Dose (mg)	0.0013	0.68
Verbal Fluency	-0.061	$<2x10^{-16}$

Influence of case status (HD vs control) on Kloppel premature responses. Pseudo R^2 = 0.92, standard error 3.30. FSIQ - full scale intelligence quotient

Supplementary Table 6d: Irritability - Kloppel Premature Response

	Estimate	P Value
(Intercept)	-0.48	0.33
Kloppel Premature Responses	-0.0025	0.89
(Intercept)	16.062	0.16
Kloppel Premature Responses	0.013	0.79
FSIQ	-0.12	0.27
Verbal Fluency	-0.16	0.089

Influence of Kloppel premature responses on Irritability status in Huntington's disease cases. Pseudo $R^2 = 0.69$, standard error 0.95. FSIQ - full scale intelligence quotient

Supplementary Table 6e: Kloppel Total Responses - Case Status

	Estimate	P Value
(Intercept)	4.69	$<2x10^{-16}$
Case Status	-0.083	0.0015
Verbal Fluency	-0.0035	0.014
(Intercept)	4.55	$<2x10^{-16}$
Case Status	-0.12	0.0075
Age	-0.00026	0.81
Gender (Male)	0.043	0.22
FSIQ	0.0025	0.16
Olanzapine Equivalent Dose (mg)	-0.064	0.0040
Fluoxetine Equivalent Dose (mg)	-0.00022	0.83
Verbal Fluency	-0.0029	0.038

Influence of case status (HD vs control) on Kloppel total responses. Pseudo $R^2 = 0.46$, standard error 2.10. FSIQ - full scale intelligence quotient

Supplementary Table 6f: Irritability - Kloppel Total Responses

	Estimate	P Value
(Intercept)	-1.38	0.085
Kloppel Total Responses	0.0086	0.25
(Intercept)	14.40	0.20
Kloppel Total Responses	0.041	0.23
FSIQ	-0.13	0.26
Verbal Fluency	-0.22	0.057

Influence of Kloppel total responses on Irritability status in Huntington's disease cases. Pseudo $R^2 = 0.75$, standard error 0.88. FSIQ - full scale intelligence quotient

Supplementary Table 7a: FNR Post Task Score - Case Status

	Estimate	P Value
(Intercept)	3.37	<2x10^{-}16
Case Status	1.0025	<2x10^{-}16
(Intercept)	1.64	1.80x10^{-7}
Case Status	0.92	<2x10^{-16}
Gender (Male)	0.075	0.22
FSIQ	0.022	5.17x10^{-12}
Olanzapine Equivalent Dose (mg)	-0.72	<2x10^{-16}
Fluoxetine Equivalent Dose (mg)	-0.0039	0.0014
Verbal Fluency	-0.015	2.65x10^{-9}

Influence of case status (HD vs control) on frustrative non-reward (FNR) post task score. Pseudo $R^2 = 0.95$, standard error 8.13. FSIQ - full scale intelligence quotient

Supplementary Table 7b: Irritability - FNR Post Task Score

	Estimate	P Value
(Intercept)	-1.39	0.024
FNR Post-Task Irritability Score	0.011	0.031

Influence of frustrative non-reward (FNR) on Irritability status in Huntington's disease cases. Pseudo $R^2 = 0.29$, standard error 1.11.

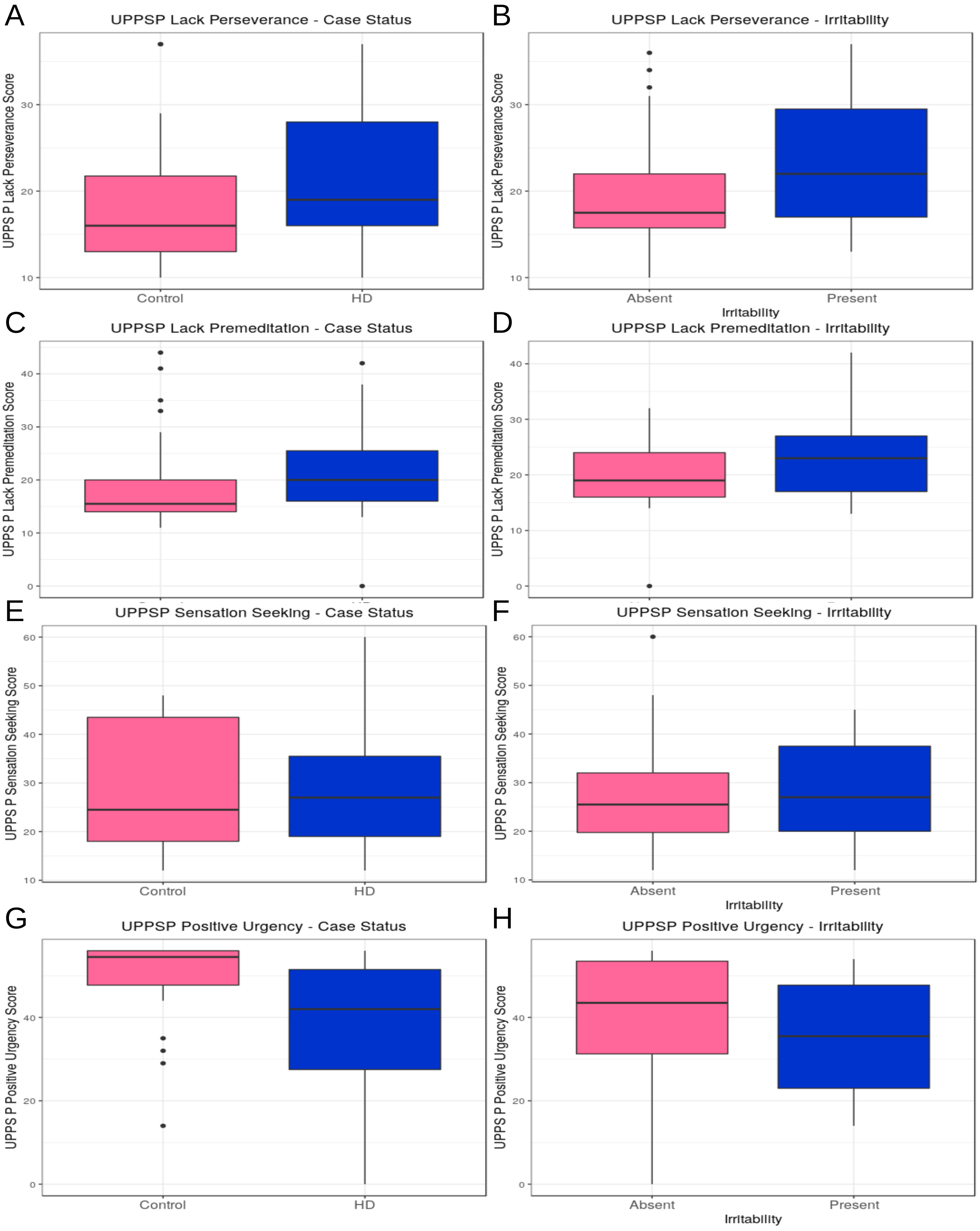

Supplementary Figure 1: UPPSP subscores. Box and whisker plots (median, interquartile range and outliers as individual points). Figures S1A,S1C,S1G – differences between HD (Huntington's disease) cases and controls. Figures S1B,S1D,S1F&S1H differences between irritable and non-irritable HD patients.

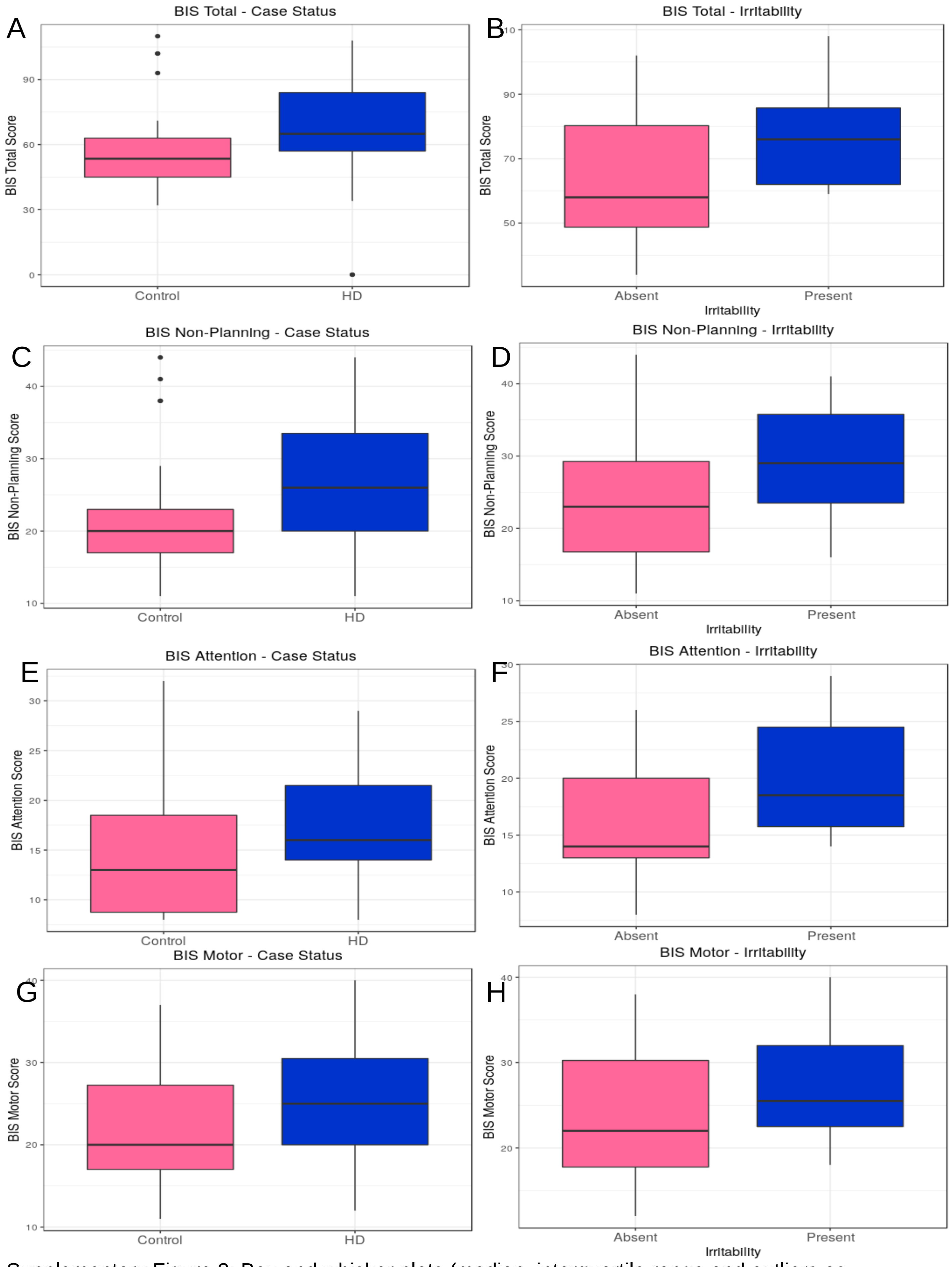

Supplementary Figure 2: Box and whisker plots (median, interquartile range and outliers as individual points) showing BIS (Barratt Impulsivity Scale) subscores. S2A,S2C,S2E,S2G, – differences between HD (Huntington’s disease) cases and controls. Figures S2B,S2D,S2F,S2H - differences between irritable and non-irritable HD patients.

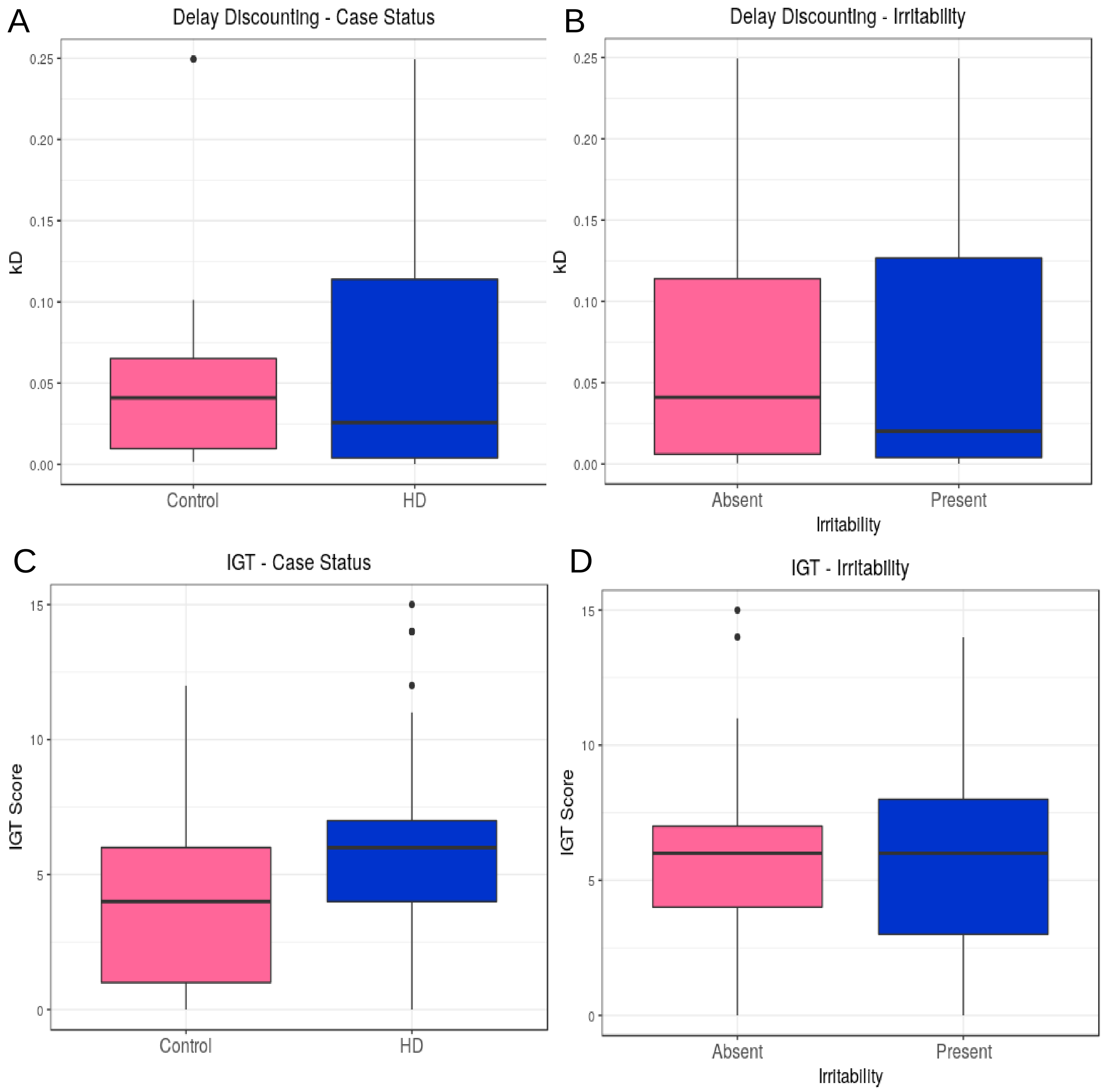

Supplementary Figure 3: Box and whisker plots (median, interquartile range and outliers as individual points) showing Impulsivity Tasks (Kirby Delay Discounting Task and Iowa Gambling Task (IGT). Figures S3A,S3C – differences between HD (Huntington's disease) cases and controls. Figures S3B,S3D differences between irritable and non-irritable HD patients.

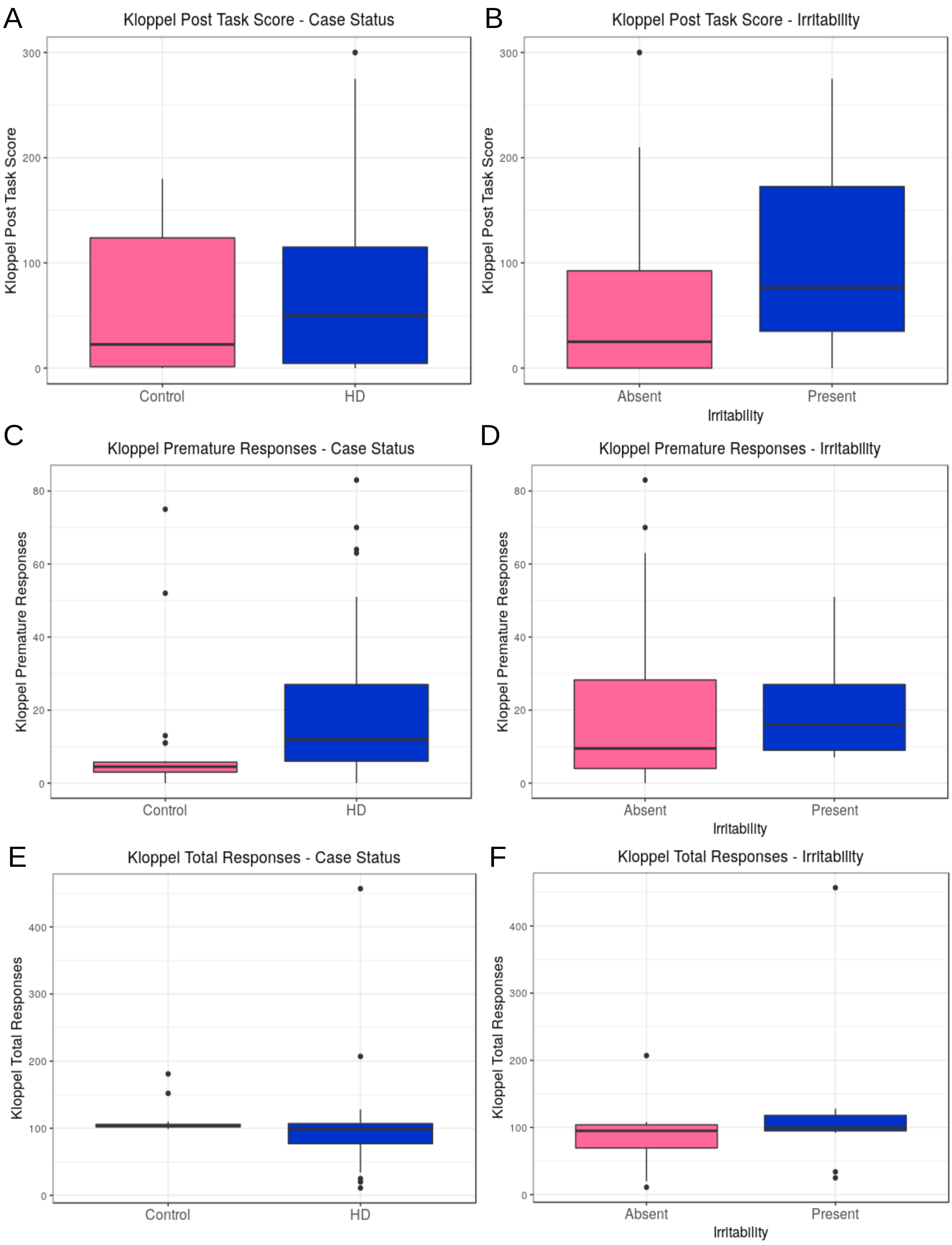

Supplementary Figure 4: Box and whisker plots (median, interquartile range and outliers as individual points) showing Kloppel Task: Post Task Scores, Premature Responses and Total Responses. Figures S4A,S4C, S4E – differences between HD (Huntington's disease cases and controls. Figures S4B, S4D, S4F differences between irritable and non-irritable HD patients.
